# New Frontiers in Molecular Imaging Using Peptide-Based Radiopharmaceuticals for Prostate Cancer

**DOI:** 10.3389/fchem.2020.583309

**Published:** 2020-12-01

**Authors:** Xin Li, Huawei Cai, Xiaoai Wu, Li Li, Haoxing Wu, Rong Tian

**Affiliations:** ^1^Department of Nuclear Medicine, West China Hospital, Sichuan University, Chengdu, China; ^2^Department of Nuclear Medicine, Frontiers Science Center for Disease-Related Molecular Network, National Clinical Research Center for Geriatrics, West China Hospital and West China School of Medicine, Sichuan University, Chengdu, China

**Keywords:** peptide, radiopharmaceuticals, prostate cancer, gastrin-releasing peptide receptor, natriuretic peptide receptor, somatostatin receptor

## Abstract

The high incidence of prostate cancer (PCa) increases the need for progress in its diagnosis, staging, and precise treatment. The overexpression of tumor-specific receptors for peptides in human cancer cells, such as gastrin-releasing peptide receptor, natriuretic peptide receptor, and somatostatin receptor, has indicated the ideal molecular basis for targeted imaging and therapy. Targeting these receptors using radiolabeled peptides and analogs have been an essential topic on the current forefront of PCa studies. Radiolabeled peptides have been used to target receptors for molecular imaging in human PCa with high affinity and specificity. The radiolabeled peptides enable optimal quick elimination from blood and normal tissues, producing high contrast for positron emission computed tomography and single-photon emission computed tomography imaging with high tumor-to-normal tissue uptake ratios. Owing to their successful application in visualization, peptide derivatives with therapeutic radionuclides for peptide receptor radionuclide therapy in PCa have been explored in recent years. These developments offer the promise of personalized, molecular medicine for individual patients. Hence, we review the preclinical and clinical literature in the past 20 years and focus on the newer developments of peptide-based radiopharmaceuticals for the imaging and therapy of PCa.

## Introduction

Prostate cancer (PCa) is the most common sex-related malignancy and second most common cause of mortality after lung cancer, accounting for 10% of all tumors in men (Siegel et al., 2020). In 2020, the American Cancer Society estimated 191,930 new cases of PCa in the United States, with PCa alone accounting for nearly 1 in 5 new diagnoses (Siegel et al., 2020). It is known that prostate tumors arise primarily in the peripheral zone (PZ) of the prostate and 20–30% often originated in the transition zone (TZ). During the early stages, patients with PCa lack specific clinical symptoms, and if they appear, the tumor is no longer resectable, and patients are at an advanced stage. PCa can be localized and advanced depending upon its metastasis *via* the lymphatic system and invade into the bones. The exact pathogenesis of PCa is still unknown, although some cases may remain ambiguous, and various factors, such as age, genetics, environmental toxins, chemical hazards, and radiations, have to be considered. Various lines of clinical evidence strongly support that proper early detection and accurate staging would significantly improve patient prognosis and the 5-year survival rate before the lesions become metastatic (DeSantis et al., 2019; Siegel et al., 2019).

Prostate-specific antigen (PSA) is generally used as an important clinical marker of early diagnosis, clinical stage, and postoperative observation for PCa because the PSA levels can rise before the presence of detectable PCa recurrence (Dall'oglio et al., 2005; Van Poppel et al., 2006). Unfortunately, this marker does not help localize and differentiate the disease. Transrectal ultrasound (TRUS) guided biopsy has been validated in prostate enhanced detection. Nearly 20% of the patients are reported to have positive results in repeated biopsies (Singh et al., 2004). Recently, interest has been growing in multiparametric magnetic resonance imaging (MP-MRI), which comprises T2-weighted sequences and several functional sequences, because no single MRI sequence adequately detects PCa (Yoo et al., 2015). Although the ideal set of sequences has not been determined, the role of MP-MRI has been expanded in PCa detection, staging, localization, and guiding biopsy. Molecular imaging plays a crucial role to detect clinically significant cancer foci. Compared with conventional PCa detection methods, such as digital rectal examination (DRE), PSA, ultrasonic imaging, and MP-MRI, targeted molecular imaging using radionuclide-labeled drugs provides not only anatomical information but also molecular, metabolic information on the whole-body scale and includes positron emission tomography/computed tomography (PET/CT) and single-photon emission computed tomography/computed tomography (SPECT/CT) (Vargas et al., 2015; Wibmer et al., 2016).

Overexpressed prostate tumor-specific receptors are detected on the cell surface and play an important role in different stages of carcinogenesis and metastasis. The classes of ligands for these receptors are peptides and their analogs, which act as important hormones, neurotransmitters, cytokines, and growth factors, and show high specificity and binding affinity to their receptors, providing a basis for targeted diagnosis and therapy. Peptides can be easily designed and produced to duplicate the critical region of the natural receptor ligand, thus achieving high specificity. With the relatively smaller structure than antibodies, peptides show rapid tissue penetration, fast clearance, low antigenicity, and easy radiolabeling ability (Ambrosini et al., 2011; Graham and Menda, 2011). Thus, radiolabeled receptor-avid peptides have been recognized as an effective strategy for molecular diagnosis and therapy in cancer treatments (Chatalic et al., 2015; Ceci et al., 2017). In this review, we summarize the newer developments of peptide-based radiopharmaceuticals for the imaging and therapy of PCa.

## Design of Peptide-Based Radiopharmaceuticals for Imaging and Therapy

**Figure 1:**
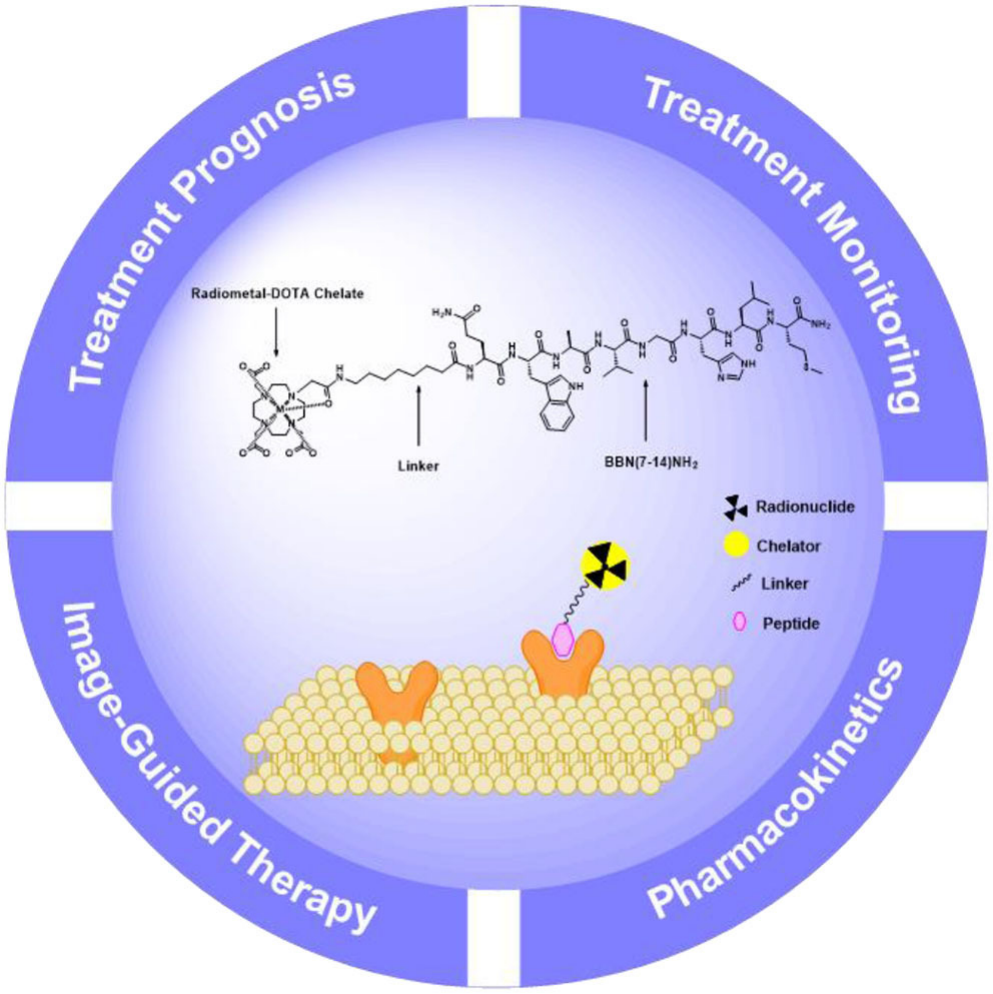
Schematic presentation of the components for peptide-based radiopharmaceuticals design.

It is known that the multi-disciplines research on molecular imaging is instructive for precise diagnosing, staging, restaging, and effective treatment of PCa. As promising molecular imaging probes, peptide-based radiopharmaceuticals have made significant progress in the fields of imaging, image-guided therapy, treatment prognosis, and treatment monitoring. Design and development of peptide-based radiopharmaceuticals for targeted molecular imaging or therapy in oncology include radionuclides with appropriate emission characteristics, metal complexing agents, pharmacokinetic modifiers, and receptor-specific targeting peptides (Figure 1). Ideally, blood clearance, receptor binding kinetics, and excretion route should be taken into account, which could affect desirable radiopeptide pharmacology (Mohtavinejad et al., 2020). For molecular imaging using radiopeptides, this is necessary in order to achieve a sufficient diagnostic signal-to-noise ratio, in an acceptable acquisition time (Sun et al., 2017). Residence time of the radiotracer in blood should be minimal, yet long enough for the agent to have its highest target uptake. It is well-established that peptide targeting ligands are beneficial in achieving high signal-to-noise ratio due to rapid renal excretion, which allowed reduction of non-specific background blood-pool and organ activity.

When choosing a radionuclide for the peptide-based radiopharmaceuticals, regardless of the strategy, there will be major considerations including the half-life, the emission profile, the method of production, and the means and ease by which the radionuclide may be chemically attached. The half-life should be appropriate enough to allow for synthesis, purification, transport, administration to the patient, and localization to target organs (Mather, 2007). These characteristics allow for optimal imaging and treatment in the half-life in a matter of a few hours. Radionuclides for peptide-based radiopharmaceuticals of PCa are listed in Table 1. Useful nuclear properties of radionuclides for optimal imaging resolution are gamma rays (γ) with energies between 100 and 200 keV for scintigraphy or SPECT imaging and positrons (β^+^) that produce annihilation radiation with an energy of a few 100 keV for PET imaging. For therapy, the effective emissions include beta minus particles (β^−^) and alpha particles (α^++^) (Stott Reynolds et al., 2018). The physical half-life of the radionuclide for a therapeutic application has to be adjusted to the retention time.

**Table 1:** Properties of radionuclides used for peptide-based radiopharmaceuticals labeling (IT, isomeric transition; EC, electron capture).

**Radionuclide**	**Abbreviations**	**Decay modes**	**Half-life**	**Emax (keV)**	**Application**
Fluorine-18	^18^F	EC, β^+^	109.8 min	635	PET imaging
Gallium-68	^68^Ga	EC, β^+^	67.7 min	1,900	PET imaging
Indium-111	^111^In	EC	2.80 d	245	SPECT imaging
Technetium-99m	^99m^Tc	IT	6.01 h	141	SPECT imaging
Yttrium-86	^86^Y	EC, β^+^	14.7 h	902	PET imaging
Copper-64	^64^Cu	EC, β^+^, β^−^	12.7 h	655	PET imaging/targeted radiotherapy
Lutetium-177	^177^Lu	β^−^	6.73 d	497	Targeted radiotherapy
Yttrium-90	^90^Y	β^−^	64 h	2,270	Targeted radiotherapy

**Figure 2:**
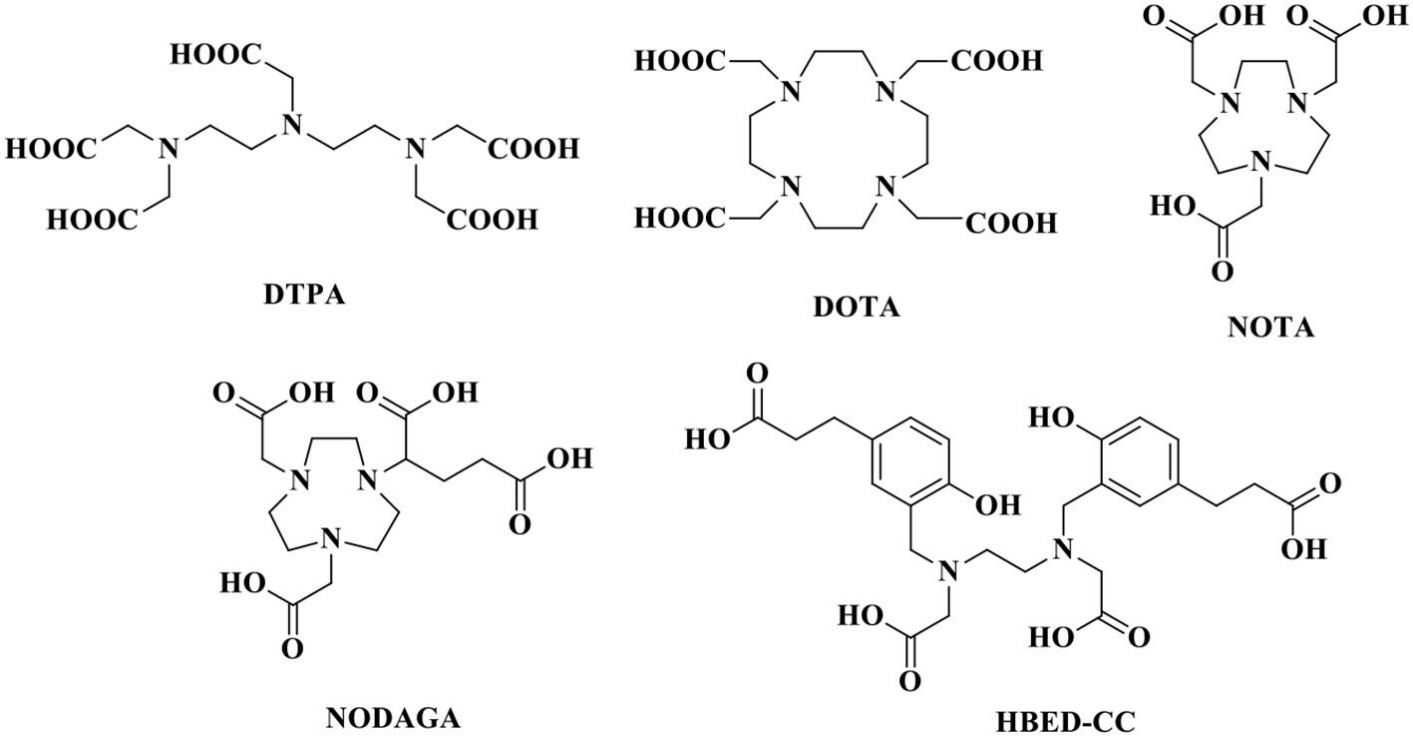
Chemical structures of typical chelators.

The stability of radiopharmaceuticals can be improved by using chelating agents for radionuclide labeling (Lucia Tornesello et al., 2017). The chemical structures of typical chelators have been shown in Figure 2. The radionuclide may disengage from the peptide without an appropriate metal chelator, resulting in excessive imaging background signal and toxicity to non-target tissues. The choice of appropriate chelators depends mostly upon the properties of the radionuclide and the ease of the conjugation chemistry (Tornesello et al., 2017). Furthermore, two broad issues clearly need to be considered, namely, kinetic and thermodynamic stability of the metal complex. Improper selection of chelating agents or poor location for attachment can decrease the binding affinity or selectivity of radiopharmaceuticals.

The linkers are commonly used as flexible linkers for the radiometal complex–peptide conjugates. The linkers by influencing the size, shape, solubility, stability, and molecular weight of the chemical structures directly support the performance of the entire radiopharmaceuticals. Moreover, the linkers affect the pharmacodynamics, affinity, and mode of excretion of the bioconjugate (Evans et al., 2020). The pharmacological behavior of radiopharmaceutical can be affected by insertion of a linking moiety as a pharmacokinetic modifier. Insertion of amino acid or aliphatic linkers between the two usually does not significantly reduce receptor binding affinity (Abbasi Gharibkandi et al., 2020). For one thing, the shorter, more polarizable linkers tend to render a more hydrophilic radioligand with primary excretion *via* the renal-urinary pathway, for another long-chained aliphatic linkers tend to produce more hydrophobic radiopeptides with unfavorably slow clearance *via* the hepatobiliary pathway. The peptide-based targeting radiopharmaceuticals can be used to detect not only the locations of the primary tumor but also the areas of metastasis, providing strong motive and helpful information for the continued development.

## Bombesin and GRPR Agonists

**Figure 3:**
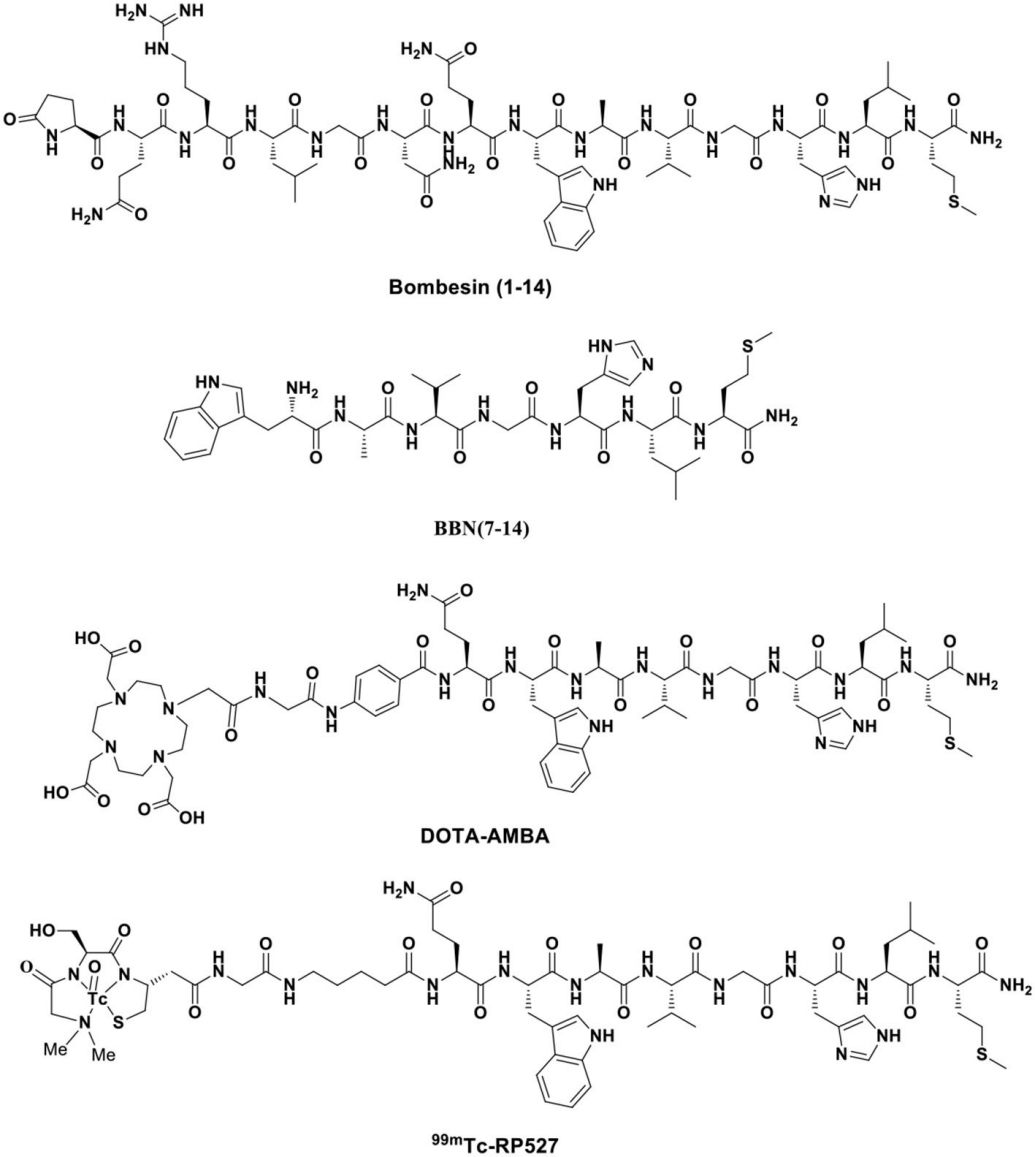
Typical bombesin and GRPR agonists developed as radiopharmaceuticals.

The amphibian bombesin (BBN) is a natural tetradecapeptide (pGlu-Gln-Arg-Leu-Gly-Asn-Gln-Trp-Ala-Val-Gly-His-Leu-Met-NH_2_) that was first isolated from the skin of the European frog *Bombina bombina* in 1971 (Figure 3). It is an analog of mammalian gastrin-releasing peptide (GRP), neuromedin B (NMB), and neuromedin C (NMC), which share a homologous 7-amino acid amidated C-terminal sequence, Trp-Ala-Val-Gly-His-Leu-Met-NH_2_ (-WAVGHLM-NH_2_) (Morgat et al., 2014; Ferreira et al., 2017). BBN is widely distributed in both peripheral tissues and the nervous system, particularly in the gastrointestinal tract (Moreno et al., 2016), and exhibits various functions by activating four subtypes of bombesin receptors (BBRs)—neuromedin B receptor (BB1R or NMBR), gastrin-releasing peptide receptor (BB2R or GRPR), orphan receptor (BB3R), and amphibious receptor (BB4R). Among the BBRs, PCa cells only express GRPR (Maffioli et al., 2015; Qiao et al., 2016). NMBR and BB3R are not expressed in PCa and are rarely found in other cancers. GRP is a regulatory peptide that plays modulatory roles in the brain, gastrointestinal tract, vascular system, and endocrine system *via* specific high-affinity GRPRs. GRP belongs to the subgroup of regulatory peptides known as bombesin-related peptides. GRP is a potent epithelial mitogen that is a 27-amino acid peptide (Ala-Pro-Val-Ser-Val-Gly-Gly-Thr-Val-Leu-Ala-Lys-Met-Try-Pro-Arg-Gly-Asn-His-Trp-Ala-Val-Gly-His-Leu-Met-NH_2_), which is essential for high specificity and affinity binding to GRPR. GRPR functions through a G protein-coupled, 7-transmembrane receptor and is present at low levels in physiologically normal organs; however, it is often overexpressed in various malignancies, including PCa, lung cancer, breast cancer, colon cancer, ovarian cancer, pancreatic cancer, renal cell cancer, and various central nervous system (CNS)/neural tumors (Smith et al., 2005; Elshafae et al., 2016). GRPR was shown to be expressed in almost 100% of prostate tumors by PCR, immunohistochemistry, and radionuclide binding assays of clinical samples (Korner et al., 2014; Accardo et al., 2016). Primary PCa often overexpresses GRPR at significantly higher levels than non-neoplastic prostate glands. GRPR expression is closely related to neoplastic transformation, cell migration, proliferation, and invasion (Varasteh et al., 2013). Preclinical data have indicated that the GRPR-positive lesion structure is histopathologically different from the surrounding GRPR-negative normal tissue. Additionally, GRPR-positive cells are polymorphic and large and have more hyperchromatic nuclei than GRPR-negative cells (Mansi et al., 2013). Therefore, using GRPR ligand analogs is an attractive strategy for the targeted molecular diagnosis and treatment of PCa.

The above results have increased the interest in bombesin analogs to image and treat PCa. In 2003, Scopinaro et al. (2003) modified the [Leu^13^] BBN(1–14) in its N-terminus to directly bind ^99m^Tc (^99m^Tc-BN) (Table 2). The literature revealed that ^99m^Tc-BN SPECT could detect PCa and positive pelvic lymph nodes. Ten patients (two with benign adenoma and eight with PCa) with disease confirmed by biopsy received 185 MBq of ^99m^Tc-BN and were subjected to SPECT. In that study, high uptake inside the prostatic fossa in all eight PCa patients was detected by true positive SPECT scans. In particular, the invasion of the obturator nodes was checked in 3 of 8 patients. Later, the team conducted another clinical trial in the PCa staging of 14 patients (De Vincentis et al., 2004). The clinical data suggested that ^99m^Tc-BN is a potential SPECT imaging candidate to detect primary PCa and loco-regional node involvement. Clinical studies have reported the use of bombesin and analogs labeled with radionuclide (^99m^Tc or ^68^Ga) to image metastasized prostate, breast, and gastrointestinal stromal tumors (Schroeder et al., 2009). In order to improve the stability of radiopharmaceuticals, the linkers and corresponding labeling methods were changed. Stabilized 4,7-lanthionine-BBN and 2,6-lanthionine-BBN peptides rapidly radiolabeled with ^18^F have been utilized. Carlucci et al. (2015) developed two novel Al^18^F-labeled BBN analogs, Al^18^F-NOTA-4,7-lanthionine-BBN and Al^18^F-NOTA-2,6-lanthionine-BBN, with good radiochemical yield (>95%) and specific activity (≥63 and ≥88 GBq/μmol) for GRPR-positive tumor imaging. In PC-3 xenograft PCa models, the tumor uptake of both radiopharmaceuticals (0.82 ± 0.23 and 1.40 ± 0.81% ID/g at 2 h p.i.) was evaluated by micro-PET (Table 3). The results showed that Al^18^F-NOTA-2,6-lanthionine-BBN might be a valuable radiotracer for human clinical translation. Chelators serve not only to chelate and stabilize radionuclides but also to tether them to other molecules *via* a chemically active functional group without causing undesirable changes in biodistribution, target binding, and pharmacokinetic. Kim et al. (2015) developed clinically translatable BBN analog-based radiopharmaceuticals for the PET imaging of GRPR-overexpressed tumors. In that study, the chelator 1,4,7-triazacyclononane, 1-glutaric acid-4,7 acetic acid (NODAGA) was used to radiolabel BBN analogs and modified galacto-BBN analogs with ^64^Cu. The preclinical studies identified that ^64^Cu-NODAGA-galacto-BBN had higher tumor uptake (3.26 ± 0.53 vs. 1.62 ± 0.23% ID/g at 1 h p.i.) and low liver uptake (1.13 ± 0.19 vs. 2.69 ± 0.69% ID/g at 1 h p.i.). These characteristics led to ^64^Cu-NODAGA-galacto-BBN as a promisingly novel PET probe for GRPR-positive PCa.

**Table 2:** Peptide-based radiopharmaceuticals targeting GRPR evaluated in human prostate cancer.

**Peptide**	**Isotope**	**Target**	**Usage**	**Dosage**	**Object**	**References**
BN (agonist)	^99m^Tc	GRPR	SPECT imaging and staging	185 MBq	10 patients with PCa and invasion of pelvic lymph nodes	Scopinaro et al., 2003
BN (agonist)	^99m^Tc	GRPR	SPECT imaging and staging	185 MBq	14 patients with primary PCa and loco-regional node involvement	De Vincentis et al., 2004
AMBA (agonist)	^68^Ga	GRPR	PET imaging and staging	160 MBq	1 patient with metastatic PCa	Baum et al., 2007
RP527 (agonist)	^99m^Tc	GRPR	SPECT imaging and staging	555 MBq	4 patients with metastatic PCa	Van de Wiele et al., 2000
RM26 (antagonist)	^68^Ga	GRPR	PET imaging and staging	51.85 MBq/kg	28 patients with primary PCa and metastasis	Zhang et al., 2018
CB-TE2A-AR06 (antagonist)	^64^Cu	GRPR	PET imaging	130–233 MBq	4 patients with newly diagnosed PCa	Wieser et al., 2014
RM2 (antagonist)	^68^Ga	GRPR	PET imaging and staging	167.9–294.5 MBq 133.2–151.7 MBq	16 patients with metastatic PCa 32 patients with BCR of PCa	Wieser et al., 2017 Minamimoto et al., 2018
SB3 (antagonist)	^68^Ga	GRPR	PET imaging and staging	283 ± 91 MBq	9 patients with advanced PCa	Maina et al., 2016
NeoBOMB1 (antagonist)	^68^Ga	GRPR	PET imaging	/	A 69-year-old patient with primary bilateral prostate adenocarcinoma	Nock et al., 2017
BAY 86-4367 (antagonist)	^18^F	GRPR	PET imaging and staging	302 ± 11 MBq	5 patients with primary PCa and 5 patients with PSA recurrence after radical prostatectomy	Sah et al., 2015
BBN-RGD	^68^Ga	GRPR	PET imaging and staging	130 MBq	5 healthy volunteers and 13 patients with PCa	Zhang et al., 2017

**Table 3:** Peptide-based radiopharmaceuticals targeting GRPR evaluated in prostate cancer, preclinical, and experimental study models.

**Radiopharmaceuticals**	**Target**	**Usage**	**Tumor**	**Tumor uptake**	**Tumor-to-muscle (T/M) ratios**	**Half time**	**References**
Al(18)F-NOTA-4,7-lanthionine-BBN Al(18)F-NOTA-2,6-lanthionine-BBN	GRPR	PET imaging	PC-3	0.82 ± 0.23% ID/g (2 h p.i.) 1.40 ± 0.81% ID/g (2 h p.i.)	9.1 ± 1.2 (2 h p.i.) 14.6 ± 1.7 (2 h p.i.)		Carlucci et al., 2015
^64^Cu-NODAGA-BBN ^64^Cu-NODAGA-galto-BBN	GRPR	Targeted radiotherapy	PC-3	1.62 ± 0.23% ID/g (1 h p.i.) 3.26 ± 0.53% ID/g (1 h p.i.)		70.7 ± 43.71 h 29.2 ± 20.61 h	Kim et al., 2015
^68^Ga-DOTA-AMBA ^177^Lu-DOTA-AMBA ^111^In-AMBA	GRPR	PET imaging Targeted radiotherapy SPECT imaging	PC-3	/ 6.35 ± 2.23% ID/g (1 h p.i.) 2.24 ± 0.66% ID/g (1 h p.i.)	/ / 3.89 (1 h p.i.)	/ / 1.53 ± 0.69 h (t_1/2α_) 30.73 ± 8.56 h (t_1/2β_)	Maddalena et al., 2009; Zhang-Yin et al., 2020 Ho et al., 2011
^64^Cu-NO2A-(8-Aoc)-BBN	GRPR	PET imaging	PC-3	3.59 ± 0.73% ID/g (1 h p.i.)			Lane et al., 2010
^68^Ga-NOTA-PEG_3_-RM26	GRPR	PET imaging	PC-3	3.31 ± 0.68% ID/g (1 h p.i.)	72.5 (1 h p.i.)		Cheng et al., 2018
^64^Cu-DOTHA_2_-PEG-RM26	GRPR	PET imaging	PC-3	4.14 ± 0.96% ID/g (1 h p.i.)			Mansour et al., 2018
68Ga-NODAGA-SCH1	GRPR	PET imaging		6.20 ± 0.53% ID/g (2 h p.i.)	16.6 ± 1.5 (2 h p.i.)		Sun et al., 2016
111In-RM1	GRPR	SPECT imaging	PC-3	14.24 ± 1.75% ID/g (1 h p.i.)	75 (1 h p.i.)		Mansi et al., 2009
Al^18^F-JMV5132 ^68^Ga-JMV5132 ^68^Ga-JMV4168	GRPR	PET imaging	PC-3	4.96 ± 1.20% ID/g (1 h p.i.) 4.73 ± 0.68% ID/g (1 h p.i.) 4.46 ± 0.33% ID/g (1 h p.i.)			Chatalic et al., 2014
^177^Lu-SB3 ^111^In-SB3	GRPR	Targeted radiotherapy SPECT imaging	PC-3	8.22 ± 1.61% ID/g (4 h p.i.) 8.78 ± 3.03% ID/g (4 h p.i.)			Maina et al., 2016; Lymperis et al., 2018
^68^Ga-NeoBOMB1 ^111^In-NeoBOMB1 ^177^Lu-NeoBOMB1	GRPR	PET imaging SPECT imaging Targeted radiotherapy	PC-3	30.7 ± 3.9% ID/g (4 h p.i.) 28.6 ± 6.0% ID/g (4 h p.i.) 42.4 ± 5.0% ID/g (4 h p.i.)			Nock et al., 2017
^18^F-AmBF3-MJ9	GRPR	SPECT imaging	PC-3	1.37 ± 0.2% ID/g (1 h p.i.)			Pourghiasian et al., 2015

To increase the tumor uptake of BBN-based radiotracers, the modification of amino acid side chains has been explored (Zhang et al., 2004). Surprisingly, AMBA (DO3A-CH_2_CO-G-(4-aminobenzoyl)-QWAVGHLM-NH_2_), a BBN-related peptide agonist, is a potent ^68^Ga-radiolabeled GRPR agonist in different malignancies (Baum et al., 2007; Zhang-Yin et al., 2020) and has been used for treatment when labeled with ^177^Lu. Maddalena et al. demonstrated that ^177^Lu-AMBA, which binds to GRPR in LNCaP and DU145 cell lines with high affinity (0.53 ± 0.10 and 0.65 ± 0.20 nM), has radiotherapeutic efficacy and decreased proliferation in LNCaP and DU145 models (Maddalena et al., 2009). However, compared with ^111^In-radiolabeled AMBA (^111^In-AMBA) (Ho et al., 2011), this tracer showed high tumor uptake (6.35 ± 2.23 vs. 2.24 ± 0.66% ID/g at 1 h p.i.) and a tumor-to-normal tissue ratio in PC-3 xenograft PCa models. The imaging of radiolabeled AMBA indicated the staging of PCa for determining the therapeutic approach and monitoring the therapeutic efficacy. Based on the C-terminal 7–14 amino acids, the peptides for GRPR imaging showed tumor localization with high specificity. More recently, substantial research efforts have been made toward backbone modification. RP527 is an octapeptide (Gln-Trp-Ala-Val-Gly-His-Leu-Met-NH_2_) derived from bombesin, and a Gly-5-Ava linker was modified to the N-terminus for tripeptide N_3_S chelator [dmgly-L-ser-L-cys(acm)] conjugation to form a stable complex with ^99m^Tc. In a clinical study, ^99m^Tc-RP527 SPECT exhibited specific tumor localization and superior imaging characteristics in four patients with metastatic PCa (Van de Wiele et al., 2000). During the same period, Van de Wiele et al. (2001) studied the biodistribution of ^99m^Tc-RP527 in GRPR-expressing human malignancies. SPECT imaging showed rapid hepatobiliary excretion and high tumor retention, resulting in high-contrast imaging and low background. To optimize the tumor-targeting properties of the radiolabeled BBN(7–14) moiety, Valverde et al. (2016) improved the metabolic stability and tumor uptake of GRPR-specific *via* radiolabeling [Nle^14^]BBN(7–14) derivatives by means of structural modifications of the peptide vector. Modifications of the C-terminus revealed that Hms (homoserine methyl ether, methoxinine) is a suitable substitute for methionine, and N-terminal variations revealed that an additional (aliphatic or aromatic) amino acid in position 6 enhanced blood plasma stability. Insertion of a 1,4-disubstituted 1,2,3-triazoles as a stable amide bond mimic at position Gly^11^-His^12^ displayed high stabilities in blood plasma (Valverde et al., 2014, 2015; Accardo et al., 2016; Maina et al., 2017a). In these studies, radiolabeling peptidotriazoles improved stability *in vitro* and increased tumor uptake *in vivo*.

Lane et al. (2010) evaluated a series of pharmacokinetic-modifying linkers of peptides based on NOTA-based BBN(7–14)-NH_2_ conjugated with ^64^Cu. In micro-PET/CT images, the eight-carbon linker showed higher tumor uptake (3.59 ± 0.73% ID/g at 1 h p.i.) and faster renal clearance (3.79 ± 1.09% ID/g at 1 h p.i.). To alleviate excessive and persistent accumulation of copper, bombesin-based imaging agents might be radiolabeled with ^68^Ga (Fischer et al., 2015; Liolios et al., 2018). Compared with ^64^Cu-NO2A-(8-Aoc)-BBN, the shorter and more hydrophilic pharmacokinetic modifier, AMBA, produced superior PET imaging with minimal accumulation in collateral tissue. The first generations of BBN analog-based radiopharmaceuticals were GRPR agonists derived from the C-terminal fragments of bombesin. GRPR agonists are internalized into tumor cells. Compared with non-modified peptides, truncated peptides present some advantages of chemical synthesis at lower cost and time. More importantly, the removal of amino acid residues increases the biological half-life and stability *in vivo* (Schottelius and Wester, 2009). Unfortunately, the rapid metabolic degradation of BBN-based radiotracers affects their tumor-targeting capability, limiting their application in medicine.

## GRPR Antagonists

Currently, various BBN analog-based radiopharmaceuticals have been used for diagnosis, staging, evaluation, and peptide receptor radionuclide therapy (PRRT) in PCa patients (Schroeder et al., 2009). Several preclinical and clinical literature sources have revealed that non-internalizing GRPR radioantagonists could successfully target and are sufficiently retained in overexpressed GRPR tumor lesions while rapidly clearing from physiologic tissues and organs (Maina et al., 2016; Baratto et al., 2018). The pharmacokinetics *in vivo* and inherent biosafety of GRPR radioantagonists are superior to their agonist counterparts (Maina and Nock, 2017; Maina et al., 2017b; Baratto et al., 2018). Compared with the corresponding radiolabeled agonist, antagonists of GRPR without internalizing or activating it were able to recognize more binding sites than agonists and had an even better tumor uptake and retention in GRPR positive tumors (Stott Reynolds et al., 2018). Current studies have been shifted from GRPR radioagonists to new improved GRPR antagonist candidates with potential clinical translation.

**Figure 4:**
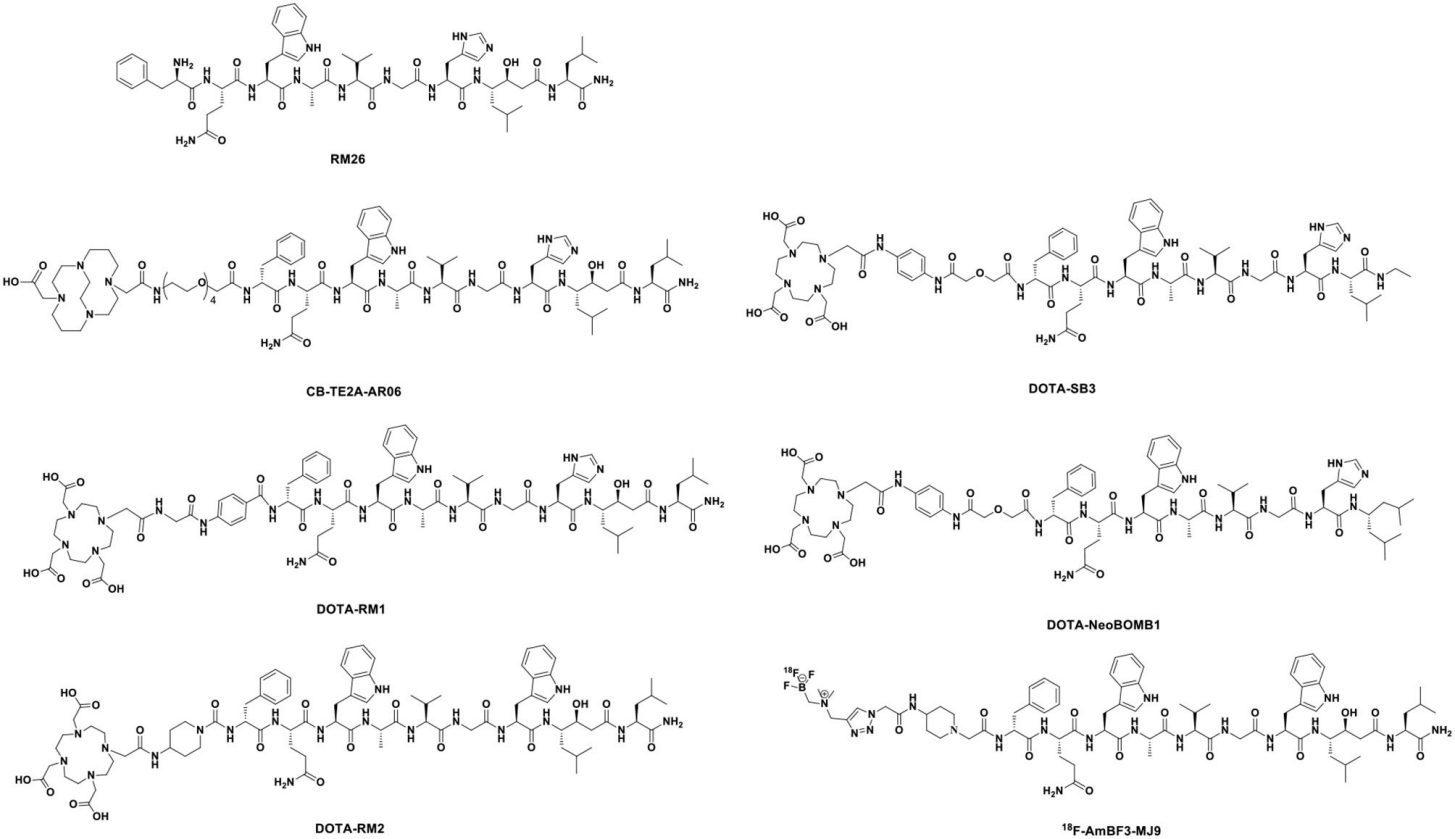
Typical GRPR antagonists developed as radiopharmaceuticals.

More recently, one of the most promising antagonists, among various GRPR antagonists, is d-Phe-Gln-Trp-Ala-Val-Gly-His-Sta-Leu-NH_2_ (RM26) for PCa imaging and treatment (Valverde et al., 2014; Zhang et al., 2018). The GRPR antagonist RM26 with high affinity was formed by the backbone modification of BBN analogs (Figure 4) (Coy et al., 1988). The application of ^68^Ga-labeled RM26 in PET/CT imaging would have remarkable value in detecting both primary PCa and metastases. Recently, another clinical study reported that ^68^Ga-RM26 PET has remarkable diagnostic value in both primary PCa and metastasis patients due to its safety and significant efficiency (Zhang et al., 2018). Generally, antagonists exhibit higher GRPR binding affinity than their corresponding agonists. Cheng et al. (2018) compared ^68^Ga-labeled BBN(7–14) and ^68^Ga-NOTA-PEG_3_-RM26 for the PET imaging of PCa. ^68^Ga-NOTA-PEG_3_-RM26, a promising PET tracer, showed higher tumor uptake (3.31 ± 0.68 vs. 2.40 ± 0.38% ID/g at 1 h p.i.) and favorable pharmacokinetics in PC-3 tumor-bearing mice. These characteristics led to high image contrast and identified RM26 as a potential antagonist for PET imaging and therapy. Changing PEG chain length had little effect on improving the tumor-to-background ratio, and PEG_2_, PEG_3_, and PEG_4_ linkers displayed similarly high tumor uptake and low kidney radioactivity uptake (Varasteh et al., 2014, 2015; Gourni et al., 2015). Additionally, this RM26 peptide showed superior characteristics for SPECT imaging when labeled with ^111^In, cobalt-55 (^55^Co), and cobalt-57 (^57^Co) (Mitran et al., 2016, 2017, 2019). Copper-64 (^64^Cu, beta^+^, Emax = 0.656 MeV; gamma, 10%; T1/2 = 16.7 h) is also an ideal positron-emitting radionuclide for PET diagnosis. More recently, Mansour et al. (2018) investigated the potential of a novel radiopharmaceutical, ^64^Cu-DOTHA_2_-PEG-RM26, to develop other ^64^Cu-labeled peptide-derived PET radioagents. In this study, ^64^Cu-DOTHA_2_-PEG-RM26 showed rapid blood clearance (from 4.42 ± 2.85% ID/g at 30 min to 0.15 ± 0.08% ID/g at 120 min), rapid renal clearance (from 8.5 ± 13.6% ID/g at 30 min to 1.03 ± 0.24% ID/g at 120 min), and specific tumor uptake (4.14 ± 0.96% ID/g at 1 h p.i.) in PC-3 tumors. This suitable biodistribution of the ^64^Cu-labeled peptides led to high contrast of PET images for clinical practice. Another novel GRPR antagonist, (^64^Cu-4,11-bis (carboxymethyl)-1,4,8,11-tetraazabicyclo(6.6.2) hexadecane)-PEG_4_-D-Phe-Gln-Trp-Ala-Val-Gly-His-Sta-Leu-NH_2_ (^64^Cu-CB-TE2A-AR06), showed very favorable characteristics, making it a promising radiopharmaceutical for PCa detection, staging, active surveillance, and radionuclide therapy. Wieser et al. (2014) reported that the PET/CT radiotracer ^64^Cu-CB-TE2A-AR06 showed favorable tumor-to-normal organ ratios in four patients with newly diagnosed PCa. Besides, SCH1 is a novel BN analog based on a modified RM26 peptide. In PC-3 cells, ^68^Ga-NODAGA-SCH1 exhibited favorable target binding affinity (3.50 ± 0.85 nM), high tumor uptake (6.20 ± 0.53% ID/g at 2 h p.i.), and high tumor/muscle contrast (16.6 ± 1.50 at 2 h p.i.). These *in vivo* imaging properties make it a potent candidate for visualized PET images. Sun et al. (2016) designed NODAGA-SCH1 radiolabeled with ^68^Ga (^68^Ga-NODAGA-SCH1) to evaluate the value for translation into the clinical PET/CT imaging of PCa patients.

The statin-based BBN analog RM26 was developed by the modification of C-terminal residues of GRPR agonists. A DOTA-Gly-benzoyl group was added to the C-terminus to form DOTA-Gly-benzoyl-d-Phe-Gln-Trp-Ala-Val-Gly-His-Sta-Leu-NH_2_ (RM1). To evaluate this antagonist as a SPECT imaging agent for GRPR, ^111^In was attached to RM1 to form ^111^In-RM1 in a rodent model. In PC-3 bearing mice, Mansi et al. (2009) reported that the antagonist RM1 demonstrated higher tumor uptake (14.24 ± 1.75% ID/g at 1 h p.i.) and more specific accumulation than the agonist in the tumors. Chatalic et al. (2014) reported three novel GRPR-targeted radiotracers, Al^18^F-JMV5132, ^68^Ga-JMV5132, and ^68^Ga-JMV4168 [JMV5132 is NODA-MPAA-βAla-βAla-[H-D-Phe-Gln-Trp-Ala-Val-Gly-His-Sta-Leu-NH_2_], JMV4168 is DOTA-βAla-βAla-[H-D-Phe-Gln-Trp-Ala-Val-Gly-His-Sta-Leu-NH_2_]]. These new PET radiotracers showed comparable high, specific accumulation (4.96 ± 1.20, 4.73 ± 0.68, and 4.46 ± 0.33% ID/g at 1 h p.i.) and fast blood clearance (0.05 ± 0.01, 0.19 ± 0.13, and 0.09 ± 0.04% ID/g at 2 h p.i.) in GRPR-positive PC-3 tumors. However, the successful application of GRPR antagonist-based peptides in nuclear imaging and therapy is hampered by their fast degradation *in vivo* with neutral endopeptidase (NEP). Interestingly, the coinjection of phosphoramidon (PA), an NEP inhibitor, may be beneficial to enhance the diagnostic sensitivity and therapeutic efficacy (Chatalic et al., 2016).

A new GRPR peptide antagonist RM2, 4-amino-1-carboxymethyl-piperidine-D-Phe-Gln-Trp-Ala-Val-Gly-His-Sta-Leu-NH_2_, was developed by Mansi et al. (2011). The antagonist RM2 has been found to have a higher affinity for GRPR than RM1 and has been labeled with ^68^Ga for PET imaging (Mansi et al., 2011). In a phase II study, the highly specific targeting of GRPR-expressing tumors and favorable pharmacokinetics have demonstrated the potential of ^68^Ga-RM2 as a prospective PET radiotracer (Iagaru, 2017). Comparison of PET/CT with histology as a gold standard indicated that ^68^Ga-RM2 has high sensitivity, specificity, and accuracy to detect, stage, and restage PCa patients (Kahkonen et al., 2013). Wieser et al. (2017) explored the diagnostic value of ^68^Ga-RM2 in a selected population of PCa patients with biochemical recurrence (BCR). In this retrospective study, ^68^Ga-RM2-PET/CT showed at least one region with focal pathological uptake in 10 of 16 patients (62.5%): 4 patients with local relapse, 4 patients with lymph node metastases, 1 patient with bone metastases, and 1 patient with lung metastasis. Additionally, PET imaging showed that ^68^Ga-RM2 has distinct biodistributions in patients with BCR of PCa (Minamimoto et al., 2016). More recently, Minamimoto et al. (2018) enrolled 32 men with BCR of PCa and observed that ^68^Ga-RM2 PET/CT identified recurrent lesions in 23 of 32 patients (detection rate = 71.8%). High uptake in multiple cancer lesions identified ^68^Ga-RM2 as a potential radiopharmaceutical for the localization of PCa patients with BCR. ^177^Lu-RM2 is suitable for the targeted treatment of metastatic castration-resistant prostate cancer (CRPC) in clinical trials. The radiopharmaceutical showed high tumor uptake and rapid clearance from normal organs (Kurth et al., 2020). RM2-PET not only showed better delineation of the gross tumor volume and distinct regions of PCa but also benefited from the information given by radiotracers targeting GRPR (Fassbender et al., 2019, 2020; Touijer et al., 2019; Baratto et al., 2020).

The PET tracer-based GRPR radioantagonist ^68^Ga-DOTA-p-aminomethyl aniline-diglycolic acid-D-Phe-Gln-Trp-Ala-Val-Gly-His-Leu-NHEt (^68^Ga-SB3) has shown excellent tumor localizing efficacy and pharmacokinetics in animals and patients with prostate and breast cancers (Maina et al., 2016; Lymperis et al., 2018). Lymperis et al. (2018) also switched ^68^Ga to the ^111^In/^177^Lu label on radiopeptides. The GRPR targeting of ^111^In- and ^177^Lu-SB3 was inferior to that of ^68^Ga-SB3 in animal models, restricting the clinical diagnostic applications of SB3. In the quest for radioligands with higher stability and potency, Nock et al. (2017) developed a novel GRPR antagonist NeoBOMB1, in which the C-terminal Leu^13^-Met^14^-NH_2_ dipeptide of SB3 to Sta^13^-Leu^14^-NH_2_ is replaced, displaying improved affinity for the GRPR and superior tumor localizing efficacy in rodent models and patients. Several studies have developed GRPR antagonist-based peptides labeled with different radionuclides, including ^67/68^Ga, ^111^In, and ^177^Lu (Dalm et al., 2017; Kaloudi et al., 2017). These NeoBOMB1-based radiopharmaceuticals, which specifically bound to the surface of PCa cells, displayed excellent tumor uptake and favorable pharmacokinetics. These beneficial features led to successful visualization in tumor lesions and high-contrast PET imaging.

PCa is one of the few neoplasms that is not well-served by conventional ^18^F-fluorodeoxyglucose (FDG) PET (Weber, 2014; Schuster et al., 2016; Bednarova et al., 2017). The new bombesin analog MJ9 (H-4-amino-1-carboxymethyl-piperidine-D-Phe-Gln-Trp-Ala-Val-Gly-His-Sta-Leu-NH_2_), which originates from GRPR antagonists with a higher binding affinity and a longer retention time, has received much attention in recent years as a promising preclinical candidate (Gourni et al., 2014). In 2015, ^18^F-AmBF3-MJ9, a novel radiofluorinated derivative of bombesin targeting GRPR, was used for PET imaging in a PC-3 PCa xenograft model (Pourghiasian et al., 2015). The tumor uptake values of ^18^F-AmBF3-MJ9 were 1.37 ± 0.2% ID/g at 1 h p.i. and 2.20 ± 0.13% ID/g at 2 h p.i. These preclinical data suggest that low background uptake and excellent tumor visualization lead to high-contrast images. Currently, ^68^Ga-NOTA-MJ9 is in phase I clinical studies. In the same year, a clinical study (Sah et al., 2015) reported the first-in-man study of the ^18^F-radiolabeled potent synthetic GRPR antagonist 3-cyano-4-^18^F-fluorobenzoyl-Ala(SO_3_H)-Ala(SO_3_H)-Ava-Gln-Trp-Ala-Val-NMeGly-His-Sta-Leu-NH_2_ (BAY 86-4367), which is used for PET imaging in patients with primary and recurrent PCa. The 10 patients (5 with primary PCa and 5 with PSA recurrence after radical prostatectomy) received intravenous administration of 302 ± 11 MBq of BAY 86-4367. The PET imaging results showed that 3 of 5 patients with primary PCa showed positive tumor delineation in the prostate, and that 2 of 5 patients with BCR showed a lesion suggestive of recurrence. The ^18^F-labeled BAY 86-4367, which successfully delineated tumors in patients, exhibited high tumor-to-background and malignant-to-normal prostate tissue ratios.

In summary, these peptide-based radiopharmaceuticals, which retain binding specificity to GRPR, demonstrated high yields, high stability, high tumor uptake, and rapid clearance of radioactivity from blood or physiologic non-GRPR-expressing organs in rodent models and humans. Beyond the above successes, many candidates of GRPR radiopeptides have demonstrated limitations in clinical research or have not yet been translated into clinical settings. For example, ^99m^Tc-HYNIC(tricine/TPPTS)-Aca-Bombesin(7–14) (^99m^Tc-HABBN) was found to be hampered by an unexpected low metabolic stability *in vivo*. ^99m^Tc-HABBN SPECT/CT imaging failed to detect PCa in patients with biopsy-proven disease (Ananias et al., 2013). As appreciation for GRPR in PCa deepens, radiotherapy and imaging using radiolabeled peptides hold great promise for PCa.

## Dual-Targeting GRPR Probes

Dual or multiple-targeting probes can recognize multiple targets, which have the advantages of better sensitivity to imaging and radiotherapy in the presence of target heterogeneity, in contrast to single-targeting probes. More recent targets of interest to αVβ3 integrin have been used to target αVβ3 receptors on tumor cells and the neovasculature. Bandara et al. (2018) developed a dual-target theranostic agent that can target GRPR and αVβ3 integrin. In that study, ^86^Y or ^90^Y-labeled RGD-Glu-[DO3A]-6-Ahx-RM2 peptide showed high binding affinity for GRPR (IC50 = 5.65 ± 0.00 nM) in PC-3 cells. The preclinical studies of RGD-Glu-(^90^Y-DO3A)-6-Ahx-RM2 identified high tumor uptake and retention of radiopharmaceuticals (8.70 ± 0.35% ID/g at 1 h p.i., 5.28 ± 0.12% ID/g at 24 h p.i.) in PC-3 tumor-bearing mice. The RGD-Glu-(^86^Y-DO3A)-6-Ahx-RM2 and RGD-Glu-(^90^Y-DO3A)-6-Ahx-RM2 matched-pair conjugates exhibited favorable pharmacokinetic profiles and improved sensitivity to detect PCa cells using micro-PET. Furthermore, ^111^In, ^64^Cu, and ^177^Lu can also be used to label these probes (Durkan et al., 2014; Stott Reynolds et al., 2015).

**Figure 5:**
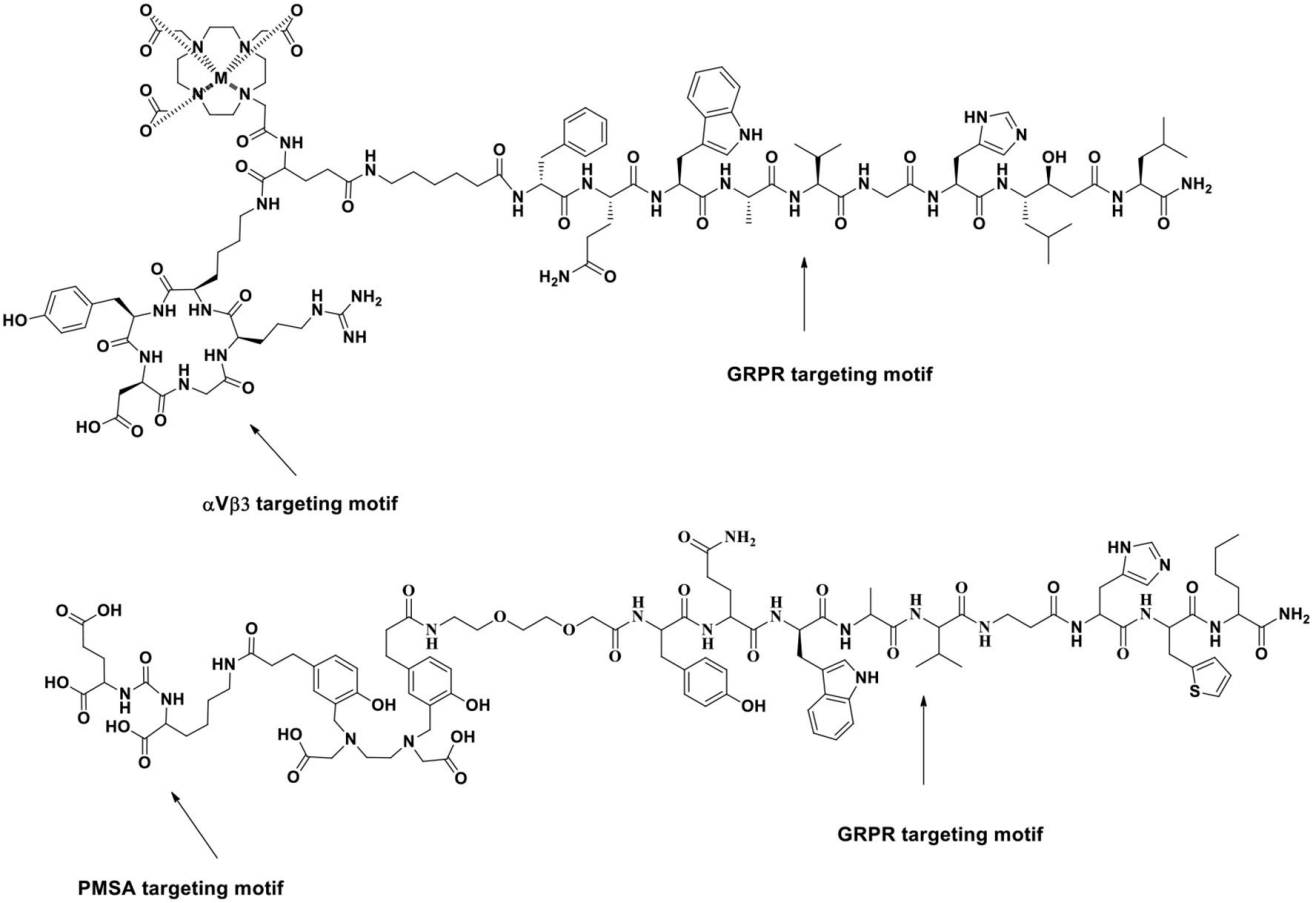
Schematic illustration of a new series of BRLs targeting PSMA and GRPR for improved PET imaging of PCa. Reprinted from Ananias et al. (2013) with permission.

In 2017, the first-in-human application of the ^68^Ga-labeled heterodimeric peptide BN-RGD targeting both αVβ3 integrin and GRPR for the clinical diagnostic imaging in PCa patients was introduced by Zhang et al. (2017) (Figure 5). In that study, 5 healthy volunteers (4 men and 1 woman) and 13 patients (4 newly diagnosed and 9 post-therapy) received a typical 130 MBq (3.5 mCi) injected dose of ^68^Ga-BBN and then were exposed to a radiation dose of 2.90 mSv with no side effects. In 13 recruited patients with PCa, ^68^Ga-BBN-RGD PET/CT detected 3 of 4 primary tumors (SUVmax = 4.46 ± 0.50), 14 metastatic lymph nodes (SUVmax = 6.26 ± 2.95), and 20 bone lesions (SUVmax = 4.84 ± 1.57). This study indicated that ^68^Ga-BBN-RGD is safe and well-tolerated, and its use in PET/CT would provide tumor staging information and a monitoring response of bone metastases in PCa patients. Moreover, Lucente et al. (2018) developed a novel ^64^Cu-radiolabeled RGD_2_-BBN heterotrimer E[c(RGDyK)]_2_-PEG_3_-Glu-(Pro-Gly)_12_-BBN(7-14)-NH_2_(RGD_2_-PG_12_-BBN) for PET imaging of PCa.

Some of the most promising PET radiotracers currently in evaluation target prostate-specific membrane antigen (PSMA), which is overexpressed in PCa tissues compared with that in normal tissues and tends to increase with the degree of aggressiveness and metastatic potential of PCa. Bandari et al. (2014) developed ^64^Cu-radiolabeled DUPA-6-Ahx-(NODAGA)-5-Ava-BBN(7–14)NH_2_, which is the first example of a dual GRPR/PSMA-targeting radioligand for the molecular imaging of prostate tumors. In 2016, Liolios et al. (2016) reported a new series of bispecific radioligands (BRLs) targeting PSMA and GRPR both expressed on PCa cells. They developed a BBN analog (H_2_N-PEG_2_-[d-Tyr^6^, β-Ala^11^, Thi^13^, Nle^14^] BBN(6–14)) that specifically binds to GRPR and a peptidomimetic urea of the pseudoirreversible PSMA inhibitor (Glu-ureido-Lys) that binds to PSMA. The two pharmacophores were linked by amino acid linkers comprising positively charged His (H) and negatively charged Glu (E) *via* copper(I)-catalyzed azide–alkyne cycloaddition to the bis (tetrafluorophenyl) ester of the chelating agent HBED-CC. The study showed that ^68^Ga-HE_n_ (*n* = 0–3) could synergistically target PSMA and GRPR on LNCaP and PC-3 PCa cells and tumor xenografts. In particular, ^68^Ga-HE_2_ improved the tumor-to-normal organ ratios in LNCaP tumor-bearing mice and significantly reduced uptake in normal organs. The bispecific targeting abilities and optimized pharmacokinetics of the BRLs could lead to further application in the clinic as sensitive radiotracers for PET/CT imaging of PCa. The radiolabeled heterobivalent peptidic ligands (HBPLs), which can bind different receptors, are highly interesting tumor imaging agents because they can improve tumor targeting for PCa in contrast to single-targeting probes. A similar study was carried out by Eder et al. (2014).

Two natural hormone peptide receptors, GRP and Y1, show upregulated expression in most breast cancer and PCa cases. Ghosh et al. (2017) developed a gadolinium-153 (^153^Gd)-labeled heterobivalent dual-targeting probe that can bind both GRP and Y1 receptors, t-BBN/BVD15-DO3A, where the GRPR ligand J-G-Abz4-QWAVGHLM-NH_2_ and Y1 receptor ligand INP-K [ε-J-(α-DO3A-ε-DGa)-K] YRLRY-NH_2_ were coupled. *In vitro*, the metabolic degradation of t-BBN/BVD15-DO3A is significantly slower than that of single-targeting probes in mouse and human serum at 37°C. The competitive displacement cell binding assay showed that the dual-target probe has strong affinity for GRP receptors in T-47D cells (IC50 value = 18 ± 0.7 nM) and Y1 receptors in MCF7 cells (IC50 value = 80 ± 11 nM). Recently, Lindner et al. (2018) reported a dual-targeting GRPR probe and vasoactive intestinal peptide receptor subtype 1 (VPAC1R) probe and radiolabeled different HBPLs comprising BBN(7–14) and PACAP-27 with ^68^Ga. The study indicated that the dual-targeting radiopharmaceuticals could bind to their target receptors, contributing to tumor cell uptake in different PCa cell lines (PC-3, DU-145, and VCaP cells).

## Somatostatin Analogs

Somatostatin is a small cyclic neuropeptide of 14 or 28 amino acids that is present in neurons and endocrine cells and functions as an inhibitive peptide with exocrine, endocrine, and autocrine activities. The inhibitory function of somatostatin on organs and tissues is likely mediated by various somatostatin receptors (SSTRs). The SSTR has five known subtypes (SSTR 1–5), and subtype 2 (SSTR 2) is thought to be most closely related to tumors. Although the multiple subtypes may be frequently located in the same cell, the binding of available somatostatin analogs to various SSTR subtypes shows significant differences.

The normal prostate comprises stromal and epithelial components. The epithelial compartment contains luminal epithelial cells, basal cells, and a few neuroendocrine cells, which regulate the growth, differentiation, and secretion of the prostate gland. In the advanced stages of PCa, the number of neuroendocrine cells increases, especially in hormonally treated and hormone-refractory (androgen-independent) PCa. The absence of neuroendocrine cells in PCa is correlated with a favorable prognosis, and neuroendocrine differentiation in tumor specimens predicts a more accurate survival rate than the Gleason score in PCa. Neuroendocrine-differentiated prostate cancer (NEPC) is an uncommon pathophysiological condition, accounting for 0.5–2.0% of all PCa cases. However, focal NEPC occurs in 10–100% of localized prostate adenocarcinomas, and the incidence increases as the disease progresses. Neuroendocrine differentiation, carrying important prognostic information, is most frequently observed during the advanced stages of PCa (Usmani et al., 2019). Due to NEPC in some PCa patients, radiolabeled somatostatin analogs are valuable for their PCa screening and therapy.

**Figure 6:**
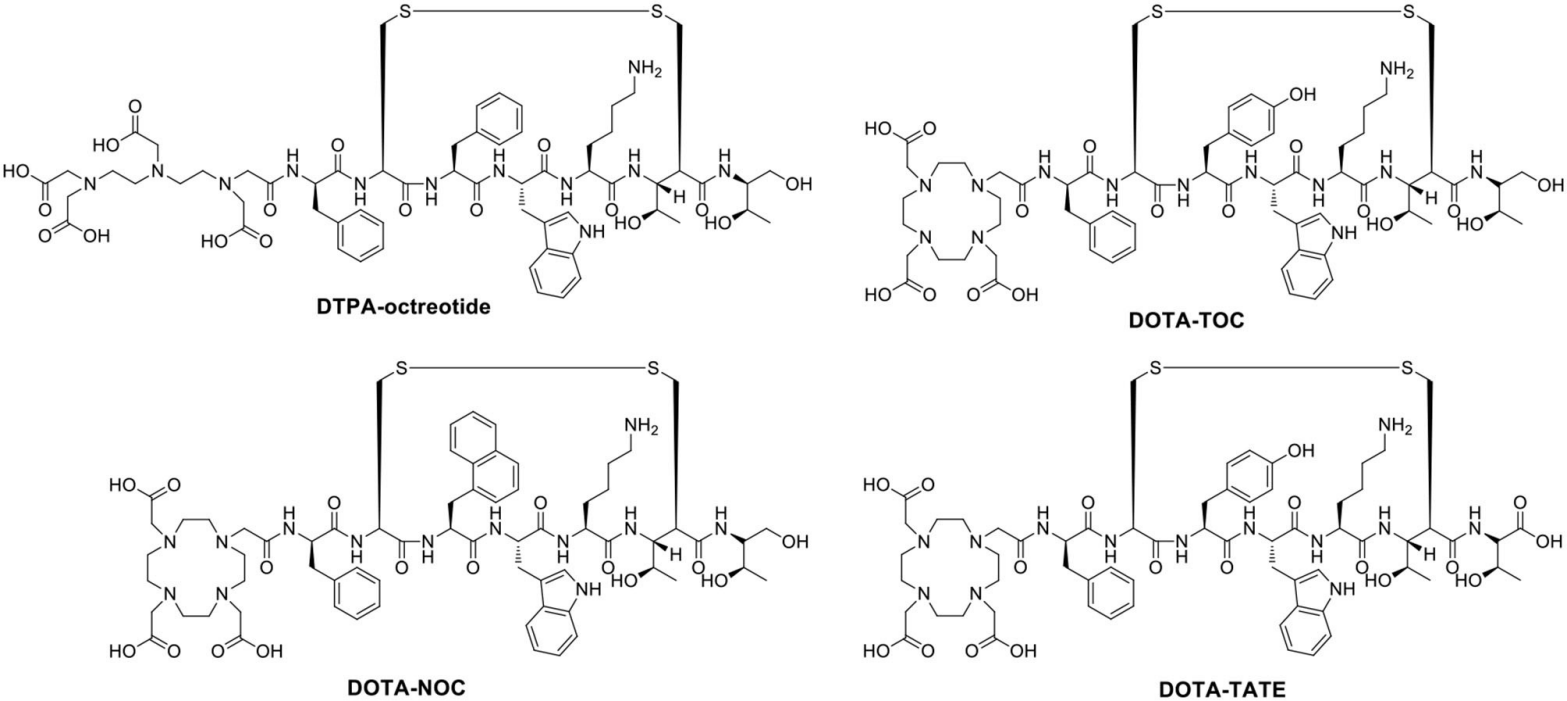
The labeled ligands of somatostatin analogs for prostate cancer diagnosis and treatment.

Naturally occurring somatostatin has very low metabolic stability *in vivo*. Accordingly, various synthetic somatostatin agonists (Figure 6) are available to study the biochemical properties of PCa with neuroendocrine differentiation, but the earliest and most widely used radiolabeled analog of somatostatin is ^111^In-DTPA-octreotide (octreoscan), which has been approved by the U.S. Food and Drug Administration (FDA) in 1994. ^111^In-DTPA-octreotide has specific high affinity for SSTR 2 and shows promising imaging applications in SSTR 2-expressing tumors (Behr et al., 2001). In a subgroup of 26 patients, octreotide scintigraphy was used to assess the neuroendocrine pattern in CRPC to select potential responders to target therapies (Table 4) (Matei et al., 2012). Additionally, Nilsson et al. (1995) reported clinical research approximately ^111^In-DTPA-octreotide imaging for metastatic hormone-refractory prostatic cancer (HRPC). Thirty-one patients were investigated scintigraphically using octreoscan, and 128 of 346 lesions confirmed by ^99m^Tc-HDP were visualized using the octreoscan technique, accounting for a 37% detection rate. The biopsies of eight patients displayed a low density of SSTRs localized on tumor cells. Notably, high uptake was detected in almost all known bone metastasis involving specific host-tissue recognitions of circulating PCa cells on ^99m^Tc-octreotide scintigraphy (which detects SSTR 2). On SSTR scintigraphy with octreotide, only 3 of 19 patients showed sites consistent with increased uptake detected by ^99m^Tc bone scintigraphy (Koutsilieris et al., 2007). In nude mice bearing experimental human PCa cells, Thakur et al. (1997) found higher uptake of a c-radiolabeled somatostatin analog. Low doses of octreoscan can also be given to visualize SSTR-positive tumors in patients with PCa. Efforts are being made to assess the efficacy of high-dose octreoscan in PCa treatment. The radiolabeled octreoscan scintigraphy was approved by the FDA for patients with neuroendocrine tumors. Since then, ^111^In-octreotide has also been used to localize and stage SSTR-positive tumors. Subsequently, clinical studies have shown that ^111^In-octreotide is promising for the treatment of various SSTR-positive tumors (Spieth et al., 2002). However, due to lower SSTR 2 expression in most prostate malignancies, diagnostic radiolabeled somatostatin analog scintigraphy or PRRT is not commonly applied for PCa patients (Liu, 2006).

**Table 4:** Peptide-based radiopharmaceuticals targeting SSTR evaluated in human prostate cancer.

**Compound**	**Isotope**	**Target**	**Usage**	**Dosage**	**Object**	**References**
DTPA-octreotide	^111^In	SSTR	SPECT imaging and staging	110–150 MBq	26 patients with CRPC 31 patients with metastatic HRPC	Matei et al., 2012 Nilsson et al., 1995
DOTA-lanreotide	^111^In	SSTR	SPECT imaging and treatment monitoring	150 MBq	1 patient with PCa (^90^Y-DOTA-lanreotide radiotherapy)	Virgolini et al., 1998
DOTA-TOC	^68^Ga	SSTR	PET imaging and staging	139 ± 26 MBq	20 patients with metastatic PCa	Luboldt et al., 2010
DOTA-NOC	^68^Ga	SSTR	PET imaging	/	A 62-year-old man with metastatic PCa (androgen blockade treatment)	Usmani et al., 2017
DOTA-TATE	^68^Ga	SSTR	PET imaging and staging	120–200 MBq 2.0 MBq/kg	12 patients with metastatic PCa 64 patients with BCR	Gofrit et al., 2017 Dos Santos et al., 2018

Compared with diethylene triamine pentaacetate acid (DTPA), the chelator 1,4,7,10-tetraazacyclododecane-1,4,7,10-tetraacetic acid (DOTA) shows higher binding affinity and better *in vivo* stability for somatostatin analogs, allowing the chelation of beta-emitting radionuclides, such as ^90^Y, for PRRT. Virgolini et al. (1998) reported the clinical use of another somatostatin analog, lanreotide, which also binds to human somatostatin receptor (hSSTR) subtypes 2–5 with high affinity. In that study, the PCa patient was administered ^111^In-DOTA-lanreotide to evaluate the response of ^90^Y-DOTA-lanreotide radiotherapy. The second somatostatin analog labeled with ^90^Y and applied to PRRT was more lipophilic. It has a high affinity for SST5 and an affinity for SST2 comparable to that of octreotide. The tumor sites were visualized after the injection of ^111^In-DOTA-lanreotide. The radiation-absorbed dose was 1.2 mGy/MBq for primary tumors and their metastatic lesions. The absorbed radiation dose of ^111^In-DOTA-lanreotide in tumors was significantly higher (ratio 2.25 ± 0.60; *P* < 0.01) than that of ^111^In-DTPA-octreotide. However, no further details about the radiotherapeutic response in this patient with PCa were discussed. During the 72-h observation period, no side effects were recorded after ^111^In-DOTA-lanreotide administration.

PET imaging of somatostatin analogs labeled with gallium-68 has shown superiority over other modalities in neuroendocrine-like tumor imaging (Kamaleshwaran et al., 2017). Previously, the radioactive imaging tracer octreoscan was extensively used in SPECT imaging to study the different biochemical properties of the differentiation of neuroendocrine tumors (di Sant'Agnese, 2001). Compared with DTPA, the chelator DOTA showed higher affinity binding for beta-emitting radionuclides and somatostatin analogs. The increased availability of ^68^Ga-radiolabeled PET tracers has allowed promising PET/CT imaging for tumors with SSTRs (Giovacchini et al., 2017). PET represents better spatial resolution and sensitivity than SPECT; thus, a series of positron-emitting radiopeptide drugs for SSTRs was developed using coordination chemistry. Gallium-68 (beta^+^, Emax = 1.92 MeV; gamma, 11%; T1/2 = 68 min) was introduced as ^68^Ga-DOTA-1-NaI^3^-octreotide (^68^Ga-DOTA-NOC) for PET/CT guiding management (Parida et al., 2018). Usmani et al. (2017) reported a 62-year-old man with metastatic PCa and androgen blockade treatment. The PSA level of the patients was only 0.049 ng/ml, the ^18^F-NaF and ^68^Ga-PSMA PET/CT scans were negative, and the ^68^Ga-DOTA-NOC scan imaged multiple somatostatin-avid hepatic and lymph node metastases. Compared with DOTA-NOC, the substitution of the DOTA-(Tyr^3^)-octreotide (DOTA-TOC) phenylalanine residue at position 3 by tyrosine increased the hydrophilicity and affinity for SST2, leading to higher uptake in SST2-positive tumors (Kwekkeboom et al., 1999). To assess the expression of DOTA-TOC-affine SSTR in advanced PCa and its bone metastases, 20 patients underwent bone scintigraphy with ^68^Ga-DOTA-TOC PET/CT (Luboldt et al., 2010). Thirteen patients had focal metastases, in which 64 of 216 metastases were visualized, and 6 patients with diffuse metastases were observed on PET imaging. Additionally, one PCa patient with a neuroendocrine metastasis showed no correlation on PET. The occurrence and localization of SSTRs in the prostate remains unclarified, and the presence of SSTRs in CRPC needs further explanation. Currently, one phase III clinically diagnostic trial was conducted to study the overexpression of SSTRs in CRPC patients by PET/CT after the injection of ^68^Ga-DOTA-NOC. The localizations of increased uptake, corresponding to metastasis detected by other methods, were considered SSTR-expressing metastases. False-positive SSTR expression with suspicious uptake could not be confirmed by other radiological examinations. Furthermore, metastatic lesions lacking the uptake of radiopharmaceuticals were considered to be non-SSTR expressing. The preliminary results showed that 2 of 6 patients had variable SSTR expression among the 67 patients with existing metastases (Savelli et al., 2015).

The somatostatin analog DOTA-(Tyr^3^)-octreotate (DOTA-TATE), in which the C-terminal threoninol is replaced with the natural amino acid threonine, has a nine-fold higher affinity for the SSTR subtype 2 than the DOTA-TOC; therefore, new radiopharmaceuticals have been developed (Acar and Kaya, 2019; Assadi and Ahmadzadehfar, 2019; Assadi et al., 2019; Dos Santos et al., 2019). Gofrit et al. (2017) used ^68^Ga-DOTA-TATE to evaluate CRPC patients with NED. Each patient received the administration of ^68^Ga-DOTA-TATE (120–200 MBq). In 12 participants, PET/CT imaging showed at least 1 patient with blastic metastasis exhibiting specific uptake of ^68^Ga-DOTA-TATE (SUVmax = 5.3 ± 2.3), 6 patients demonstrating moderately high uptake (SUVmax >5), and 4 patients with lytic bone lesions or lymph node metastases indicating uptake of the tracer (SUVmax = 7.2 ± 3.2). Patients with multiple bone metastases had significantly higher SUVmax values than those with few metastases (SUVmax 5.8 vs. 3.8, *P* = 0.05). Prospective comparison of the detection rates of ^68^Ga-DOTA-TATE and ^11^C-choline PET/CT in PCa patients with BCR was used to evaluate the targeted therapy and diagnosis of SSTR expression. Dos Santos et al. (2018) analyzed 64 patients with BCR and found that the total detection rates per patient were 48.43% for ^68^Ga-DOTA-TATE and 46.87% for ^11^C-choline. The SUVmax of ^11^C-choline was higher than that of ^68^Ga-DOTA-TATE, whereas the sensitivity and specificity values per patient were the same. Moreover, DOTA-TATE labeling with the beta- and gamma-emitting radionuclide lutetium-177 (^177^Lu) demonstrated favorable advantages for radiotherapy regarding the tumor extinction and survival period in a rat model (Breeman et al., 2003). Additionally, another cyclic somatostatin analog, RC-160, was successfully labeled with rhenium-188 (^188^Re), a beta- and gamma-emitting radionuclide (beta-, Emax = 2.12 MeV; gamma-, 10%; T1/2 = 16.7 h), and was evaluated in nude mice bearing xenografts of human prostate adenocarcinoma for its therapeutic potential by Zamora et al. (1996, 1997). ^188^Re-RC-160 was selectively retained in both DU-145 and PC-3 tumors following direct intratumor injection over 24 h, indicating a dose-dependent therapeutic response. However, scant literature exists about this cyclic peptide in recent decades, and no translational trials have been reported (Bender et al., 1997). All clinically relevant agonists tested so far (octreotide, lanreotide, and vapreotide) are selective for SSTR 2, less potent for SSTR 3/SSTR 5, and inactive for SSTR 1 and SSTR 4. Therefore, radioactive somatostatin analogs might be useful in screening and subsequent treatment of NEPC.

## Natriuretic Peptide Analogs

Natriuretic peptides, including atrial natriuretic peptide (ANP), brain natriuretic peptide (BNP), and type-C natriuretic peptide (CNP), constitute a family of distinctly genetic hormone peptides that share common cell surface membrane receptors. Normally, ANP and BNP are generated by atrial and ventricular myocytes, whereas CNP is secreted from the vascular endothelium and male genital glands. Most of the physiological effects of natriuretic hormone peptides are mediated by the interaction with the specific high-affinity natriuretic peptide receptor A (NPR-A) and low-affinity natriuretic peptide receptor C (NPR-C) on the cell surface. The existence of NPR-A in PCa cells was first confirmed in 2005. Wang et al. (2011) demonstrated that NPR-A is overexpressed on tumorigenic mouse and human PCa cells. In human PCa tissues, NPR-A expression showed a positive correlation with clinical staging. Moreover, the apoptosis of PCa cells was induced by the downregulation of NPR-A with an NPR-A inhibitor (iNPR-A). For example, TRAMP-C1 xenografts failed to grow in mice implanted with PCa cells lacking NPR-A. After iNPR-A treatment, the tumor burden and expression of macrophage migration inhibitory factor (MIF) in mice were reduced. Collectively, these results suggest that NPR-A, which partly promotes PCa development, is a potential prognostic and therapeutic target for PCa. NPR-C accounts for a large population (>95%) of all natriuretic peptide receptors. Interestingly, NPR-C has also been identified in PCa cells, and its gene expression in a mouse xenograft model has also been confirmed. Therefore, imaging the upregulation of NPR-C could determine a powerful target for early detection in a PCa model.

**Figure 7:**
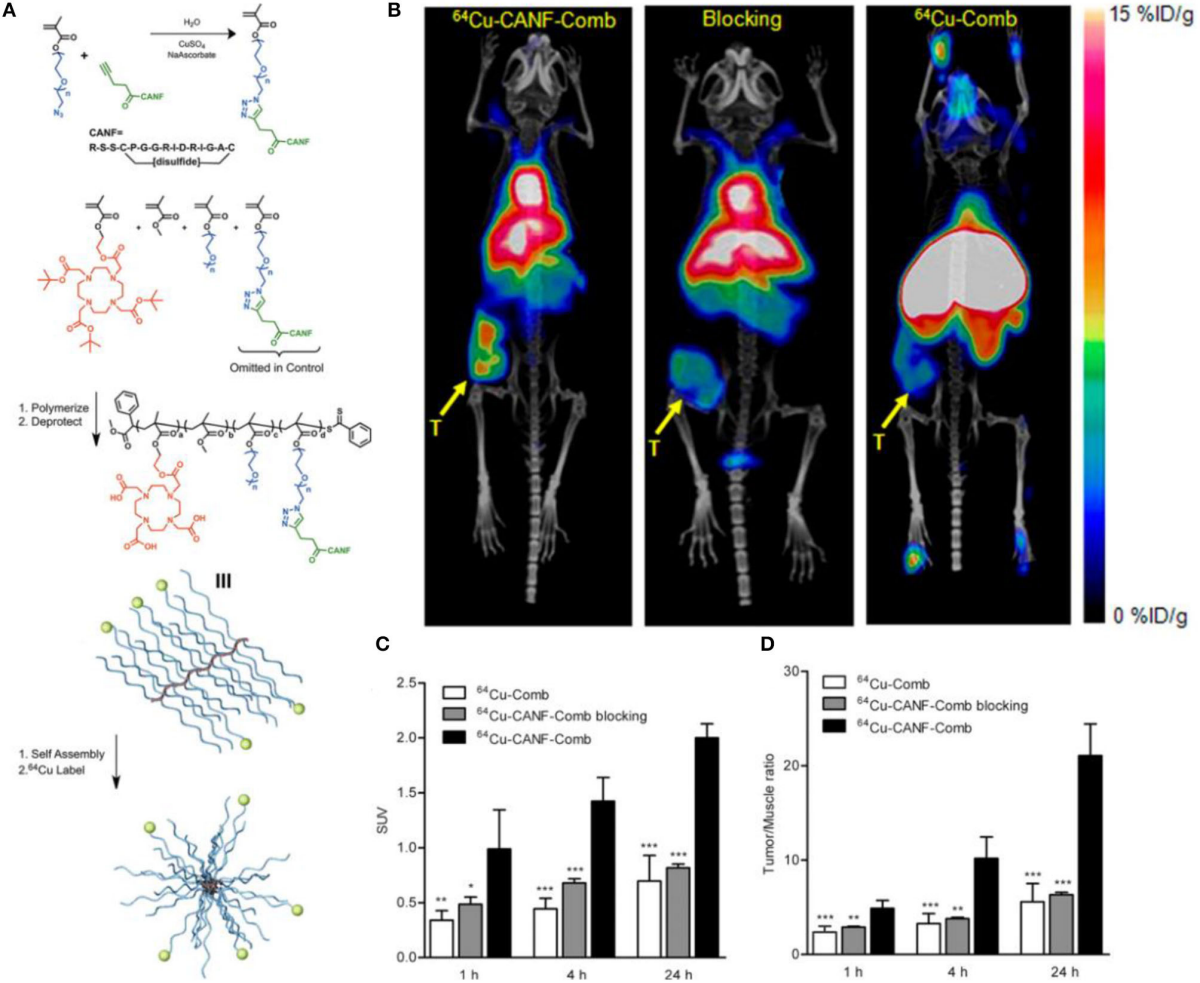
**(A)** Synthesis and subsequent assembly into nanoparticles. **(B)** PET/CT imaging of ^64^Cu-CANF-Comb, ^64^Cu-CANF-Comb blocking, and ^64^Cu-Comb in a CWR 22 tumor at 24 h p.i. **(C)** Quantitative tumor uptake SUVs and **(D)** tumor/muscle ratios of ^64^Cu-Comb, ^64^Cu-CANF-Comb, and ^64^Cu-CANF-Comb blocking in a CWR 22 tumor. Reprinted from Pressly et al. (2013) with permission.

Currently, the most common radiopharmaceutical for PET/CT imaging in the clinic, ^18^F-fluoro-2-deoxy-2-D-glucose (^18^F-FDG), is not effective in the diagnosis and localization of PCa because of its glucose-independent metabolism. Other radiotracers used for clinical research, such as ^18^F/^11^C-choline and ^11^C-acetate, have shown fast renal clearance and low tumor uptake, which may limit the potential for translational research. Moreover, none of these radiotracers are PCa specific. Nanoparticles, owing to the unique physicochemical properties, such as pharmacokinetics, targeting efficiency and specificity, have been widely used for oncological diagnosis and therapy including PCa. In a preclinical study, Pressly et al. (2013) reported that the presence of NPR-C in PCa tissues, confirmed by immunohistochemistry staining, has been exploited to establish a new nanoagent for PET imaging by synthesizing an amphiphilic comb-like nanoparticle containing C-atrial natriuretic factor (CANF) with copper-64 (^64^Cu) radiolabeling for NPR-C targeting (Figure 7). Compared with the non-targeted ^64^Cu-Comb nanoparticles, the pharmacokinetics of ^64^Cu-CANF-Comb showed a superior biodistribution profile and an optimized *in vivo* profile. In the CWR22 PCa tumor mode, PET imaging showed that ^64^Cu-CANF-Comb is characterized by high blood pool retention, low renal clearance, enhanced tumor uptake, and decreased hepatic burden in contrast to the non-targeted ^64^Cu-Comb. Compared with the other reported PCa PET radiotracers, ^64^Cu-CANF-Comb showed a sufficient T/M ratio (21.0 ± 3.4, *n* = 6–8) and a higher T/K ratio (2.3 ± 0.2, *n* = 6–8) at 24 h. Thus, ^64^Cu-CANF-Comb, which targets NPR-C *in vivo* with high sensitivity and specificity, may be a candidate for PCa PET imaging.

## Other Peptides

Other regulatory peptide analogs have the potential for radiolabeling and indicate advantages in PCa imaging and therapy. Neurotensins (NTs) are secreted by neuroendocrine-like prostate cells and demonstrate various physiological effects. Recently, overexpressed neurotensin receptors (NTRs) were suggested to be responsible for PCa proliferation and progression. Importantly, neurotensin receptor 1 (NTR 1) was found to be expressed and activated in aggressive PCa cells but not in normal cells (Taylor et al., 2012). Thus, the high incidence of NTR 1 in PCa makes it a promising target for therapy and prognosis. Neurotensin-derived factors act on PCa cells to stimulate pro-survival signaling and describe a novel mechanism of crosstalk between NE-derived factors and insulin-like growth factor 1 (IGF-1R) (DaSilva et al., 2013). Radiotherapy focusing on NTR 1 in PCa has become potentially important components of PCa treatment strategies. Deng et al. (2017) studied the PET imaging of PCa models using ^64^Cu-AmBaSar-NT, ^64^Cu-DOTA-NT, and ^64^Cu-NOTA-NT, and all the ^64^Cu-labeled neurotensin analogs retained high tumor uptake in PC-3 xenografts.

Androgen receptor (AR) and its regulated physiological pathways are central to the initiation and progression of PCa. As a member of the nuclear steroid receptor family and a transcription factor, its structure has three different functional domains—the ligand-binding domain (LBD), DNA-binding domain (DBD), and transactivation domain (TAD). Due to various factors, the clinical drugs targeting the DBD and TAD have not yet been approved, whereas all clinically active drugs against PCa target the AR-LBD (Elshan et al., 2018). Importantly, the activity of AR correlates with the growth of CRPC (Coutinho et al., 2016). Consequently, the ligands interacting with AR are potentially diagnostic and are therapeutic targets for both hormone-dependent diseases and CRPC. As a seminal plasma protein, semenogelin I (SgI) functions as an AR coactivator. At the same time, the LxxLL (L = leucine, x = any amino acid) motif in AR coregulators has been proven to mediate specific interactions with AR. Li et al. (2018) found that the LxxLL motif of SgI may be a promising therapeutic target for both androgen-sensitive cancer and CRPC. Moreover, the peptide B-8R killed both androgen-dependent and -independent PCa cells expressing soluble guanylyl cyclase α1 (sGCα1), which promoted the survival and growth of PCa cells and induced the apoptosis of PCa cells. Thus, the peptide B-8R may be a potential target against PCa cells (Zhou et al., 2017). The calcitonin peptide family plays a critical role in PCa and its bone metastasis (Warrington et al., 2017). A recent study showed convincing evidence for the important role of calcitonin peptides and receptors in PCa and as a promising prognostic target (Aljameeli et al., 2016, 2017).

The fast-increasing use of PET with PSMA ligand for the imaging of BCR has led to changes in radionuclide therapy. Importantly, PSMA is overexpressed on the surface of cells and is positively associated with PCa progression (Silver et al., 1997; Chang, 2004). PSMA is a 100-kDa type II transmembrane glycoprotein belonging to the M28 peptidase family that binds to its ligands as a dimer before internalization through clathrin-coated pits on the cellular membrane. The structure and function of PSMA are homologous to those of glutamate carboxypeptidase, such as N-acetyl-l-aspartyl-l-glutamate (NAAG). Early studies of PSMA inhibitors focused on the peptide analogs of NAAG. Unfortunately, most of these analogs showed low affinity in preclinical and clinical research (Lütje et al., 2015; Diao et al., 2019). Among the PSMA ligands, the small-molecular inhibitor, which shows excellent affinity and sufficient specificity to PSMA, exhibits the most promising effects for PCa diagnosis and treatment (Lenzo et al., 2018).

## Conclusions and Future Perspectives

Great efforts have been made in the development of peptide-based radiopharmaceuticals for PCa diagnosis and radiotherapy. With the help of coordination chemistry and newly designed chelators, many radiopeptides have been developed. Promising results in preclinical studies have been obtained regarding the biodistribution, tumor-to-organ ratio, ideal imaging contrast, and therapeutic response. The most effective members were carefully selected for clinical studies and produced high contrast and sensitivity for SPECT/CT or PET/CT imaging, as well as provided a valuable therapeutic index for PRRT in PCa.

In the past few decades, researches focusing on the development of radiolabeled peptides for PCa have been in the ascendant, but only a few radiopharmaceuticals have been translated into clinical trials. Desirable peptide-based targeting radiopharmaceuticals can bind special target receptors with high affinity, be quickly cleared from the blood and non-target tissues, and have minimal immunogenicity. Unfortunately, the drawback of targeting peptides may be that the metabolic stability needs to be improved. Currently, the peptide-based radiopharmaceuticals have ushered in a new prospect of increasingly sensitive and specific molecular targeting for PCa patients with primary and metastatic lesions, but the binding affinity, stability *in vivo*, and pharmaceutical kinetics of radiolabeled peptides are still needed to be improved. With the emergence of newly developed peptide analogs, radiotherapy using radiolabeled peptides holds great promise in the treatment of cancer, especially when used in conjunction with other therapy modalities in the future.

Because of the non-specificity and heterogenous nature of PCa, the diagnosis and therapy of PCa is one of the most challenging tasks facing a large gap. In the past 40 years, precision molecular imaging *via* radiopharmaceutical has addressed primary medical gaps, including the early diagnosis of small lesions and specific biomarker expression. In particular, the dynamic changes in biomarkers were monitored for high-risk patients during disease progression and therapy. In summary, these developments in peptide-based imaging and therapy might be useful to better understand the nature of PCa and contribute to designing more sensitive and accurate imaging agents to detection and evaluate PCa and help physicians select optimal therapy plans.

## Author Contributions

XL wrote the manuscript. HC added valuable comments to the manuscript. XW and LL made the figures and added information to the table. HW and RT critically revised and improved the manuscript. All authors have contributed to the work and approved the final version of the manuscript.

## Conflict of Interest

The authors declare that the research was conducted in the absence of any commercial or financial relationships that could be construed as a potential conflict of interest.

## Abbreviations

PCa: prostate cancer
PSA: prostate-specific antigen
PET: positron emission tomography
SPECT: single-photon emission computed tomography
CT: computed tomography
BBR: bombesin receptor
BBN: pGlu-Gln-Arg-Leu-Gly-Asn-Gln-Trp-Ala-Val-Gly-His-Leu-Met-NH_2_
GRPR: gastrin-releasing peptide receptor
GRP: Ala-Pro-Val-Ser-Val-Gly-Gly-Thr-Val-Leu-Ala-Lys-Met-Try-Pro-Arg-Gly-Asn-His-Trp-Ala-Val-Gly-His-Leu-Met-NH_2_
NODAGA: 1,4,7-triazacyclononane, 1-glutaric acid-4,7 acetic acid
DOTA: 1,4,7,10-tetraazacyclododecane-1,4,7,10-tetraacetic acid
DTPA: diethylene triamine pentaacetate acid
AMBA: (DO3A-CH2CO-G-(4-aminobenzoyl)-QWAVGHLM-NH_2_)
RP527: Gln-Trp-Ala-Val-Gly-His-Leu-Met-NH2
RM26: d-Phe-Gln-Trp-Ala-Val-Gly-His-Sta-Leu-NH_2_
RM1: Gly-benzoyl-d-Phe-Gln-Trp-Ala-Val-Gly-His-Sta-Leu-NH_2_
RM2: 4-amino-1-carboxymethyl-piperidine-D-Phe-Gln-Trp-Ala-Val-Gly-His-Sta-Leu-NH_2_
SB3: p-aminomethyl aniline-diglycolic acid-D-Phe-Gln-Trp-Ala-Val-Gly-His-Leu-NHEt
MJ9: H-4-amino-1-carboxymethyl-piperidine-D-Phe-Gln-Trp-Ala-Val-Gly-His-Sta-Leu-NH_2_
PSMA: prostate-specific membrane antigen
SSTR: somatostatin receptor
DOTA-NOC: DOTA-1-NaI3-octreotide
DOTA-TOC: DOTA-(Tyr3)-octreotide
DOTA-TATE: DOTA-(Tyr3)-octreotate
HRPC: hormone-refractory prostatic cancer
NPR: natriuretic peptide receptor
AR: androgen receptor.

## References

[B1] Abbasi Gharibkandi N. Conlon J. M. Hosseinimehr S. J. (2020). Strategies for improving stability and pharmacokinetic characteristics of radiolabeled peptides for imaging and therapy. Peptides 133:170385. 10.1016/j.peptides.2020.170385 32822772

[B2] Acar E. Kaya G. (2019). 18F-FDG, 68Ga-dotatate and 68Ga-PSMA positive metastatic large cell neuroendocrine prostate tumor. Clin. Nucl. Med. 44, 53–54. 10.1097/RLU.0000000000002322 30325820

[B3] Accardo A. Galli F. Mansi R. Del Pozzo L. Aurilio M. Morisco A.. (2016). Pre-clinical evaluation of eight DOTA coupled gastrin-releasing peptide receptor (GRP-R) ligands for *in vivo* targeting of receptor-expressing tumors. EJNMMI Res. 6:17. 10.1186/s13550-016-0175-x 26897133 PMC4761355

[B4] Aljameeli A. Thakkar A. Shah G. (2017). Calcitonin receptor increases invasion of prostate cancer cells by recruiting zonula occludens-1 and promoting PKA-mediated TJ disassembly. Cell Signal. 36, 1–13. 10.1016/j.cellsig.2017.04.008 28428082

[B5] Aljameeli A. Thakkar A. Thomas S. Lakshmikanthan V. Iczkowski K. A. Shah G. V. (2016). Calcitonin receptor-zonula occludens-1 interaction is critical for calcitonin-stimulated prostate cancer metastasis. PLoS ONE 11:e0150090. 10.1371/journal.pone.0150090 26934365 PMC4775073

[B6] Ambrosini V. Fani M. Fanti S. Forrer F. Maecke H. R. (2011). Radiopeptide imaging and therapy in Europe. J. Nucl. Med. 52(Suppl 2), 42s−55s. 10.2967/jnumed.110.085753 22144555

[B7] Ananias H. J. Yu Z. Hoving H. D. Rosati S. Dierckx R. A. Wang F.. (2013). Application of (99m)technetium-HYNIC(tricine/TPPTS)-aca-bombesin(7-14) SPECT/CT in prostate cancer patients a first-in-man study. Nucl. Med. Biol. 40, 933–938. 10.1016/j.nucmedbio.2013.05.009 23891351

[B8] Assadi M. Ahmadzadehfar H. (2019). (177)Lu-DOTATATE and (177)Lu-prostate-specific membrane antigen therapy in a patient with advanced metastatic radioiodine-refractory differentiated thyroid cancer after failure of tyrosine kinase inhibitors treatment. World J. Nucl. Med. 18, 406–408. 10.4103/wjnm.WJNM_112_18 31933557 PMC6945353

[B9] Assadi M. Pirayesh E. Rekabpour S. J. Zohrabi F. Jafari E. Nabipour I.. (2019). 177Lu-PSMA and 177Lu-dotatate therapy in a patient with metastatic castration-resistant prostate cancer and neuroendocrine differentiation. Clin. Nucl. Med. 44, 978–980. 10.1097/RLU.0000000000002824 31689280

[B10] Bandara N. Stott Reynolds T. J. Schehr R. Bandari R. P. Diebolder P. J. Krieger S.. (2018). Matched-pair, (86)Y/(90)Y-labeled, bivalent RGD/bombesin antagonist, [RGD-Glu-[DO3A]-6-Ahx-RM2], as a potential theranostic agent for prostate cancer. Nucl. Med. Biol. 62–63, 71–77. 10.1016/j.nucmedbio.2018.06.001 29929115 PMC6072280

[B11] Bandari R. P. Jiang Z. Reynolds T. S. Bernskoetter N. E. Szczodroski A. F. Bassuner K. J.. (2014). Synthesis and biological evaluation of copper-64 radiolabeled [DUPA-6-Ahx-(NODAGA)-5-Ava-BBN(7-14)NH2], a novel bivalent targeting vector having affinity for two distinct biomarkers (GRPr/PSMA) of prostate cancer. Nucl. Med. Biol. 41, 355–363. 10.1016/j.nucmedbio.2014.01.001 24508213 PMC4041584

[B12] Baratto L. Duan H. Laudicella R. Toriihara A. Hatami N. Ferri V.. (2020). Physiological (68)Ga-RM2 uptake in patients with biochemically recurrent prostate cancer: an atlas of semi-quantitative measurements. Eur. J. Nucl. Med. Mol. Imaging 47, 115–122. 10.1007/s00259-019-04503-4 31478089

[B13] Baratto L. Jadvar H. Iagaru A. (2018). Prostate cancer theranostics targeting gastrin-releasing peptide receptors. Mol. Imaging Biol. 20, 501–509. 10.1007/s11307-017-1151-1 29256046 PMC7469490

[B14] Baum R. Prasad V. Mutloka N. Frischknecht M. Maecke H. Reubi J. (2007). Molecular imaging of bombesin receptors in various tumors by Ga-68 AMBA PET/CT: first results. J. Nucl. Med. 48:79

[B15] Bednarova S. Lindenberg M. L. Vinsensia M. Zuiani C. Choyke P. L. Turkbey B. (2017). Positron emission tomography (PET) in primary prostate cancer staging and risk assessment. Transl. Androl. Urol. 6, 413–423. 10.21037/tau.2017.03.53 28725583 PMC5503952

[B16] Behr T. M. Gotthardt M. Barth A. Behe M. (2001). Imaging tumors with peptide-based radioligands. Q. J. Nucl. Med. 45, 189–200. 11476170

[B17] Bender H. Zamora P. O. Rhodes B. A. Guhlke S. Biersack H. J. (1997). Clinical aspects of local and regional tumor therapy with 188Re-RC-160. Anticancer Res. 17, 1705–1712. 9179223

[B18] Breeman W. A. Mearadji A. Capello A. Bernard B. F. van Eijck C. H. Krenning E. P.. (2003). Anti-tumor effect and increased survival after treatment with [177Lu-DOTA0,Tyr3]octreotate in a rat liver micrometastases model. Int. J. Cancer 104, 376–379. 10.1002/ijc.10952 12569562

[B19] Carlucci G. Kuipers A. Ananias H. J. de Paula Faria D. Dierckx R. A. Helfrich W. Rink R.. (2015). GRPR-selective PET imaging of prostate cancer using [(18)F]-lanthionine-bombesin analogs. Peptides 67, 45–54. 10.1016/j.peptides.2015.03.004 25797109

[B20] Ceci F. Castellucci P. Cerci J. J. Fanti S. (2017). New aspects of molecular imaging in prostate cancer. Methods 130, 36–41. 10.1016/j.ymeth.2017.07.009 28711565

[B21] Chang S. S. (2004). Overview of prostate-specific membrane antigen. Rev. Urol. 6, S13–S18. 16985927 PMC1472940

[B22] Chatalic K. L. Franssen G. M. van Weerden W. M. McBride W. J. Laverman P. de Blois E.. (2014). Preclinical comparison of Al18F- and ^68^Ga-labeled gastrin-releasing peptide receptor antagonists for PET imaging of prostate cancer. J. Nucl. Med. 55, 2050–2056. 10.2967/jnumed.114.141143 25413139

[B23] Chatalic K. L. Konijnenberg M. Nonnekens J. de Blois E. Hoeben S. de Ridder C.. (2016). *In vivo* stabilization of a gastrin-releasing peptide receptor antagonist enhances PET imaging and radionuclide therapy of prostate cancer in preclinical studies. Theranostics 6, 104–117. 10.7150/thno.13580 26722377 PMC4679358

[B24] Chatalic K. L. Kwekkeboom D. J. de Jong M. (2015). Radiopeptides for imaging and therapy: a radiant future. J. Nucl. Med. 56, 1809–1812. 10.2967/jnumed.115.161158 26514175

[B25] Cheng S. Lang L. Wang Z. Jacobson O. Yung B. Zhu G.. (2018). Positron emission tomography imaging of prostate cancer with Ga-68-labeled gastrin-releasing peptide receptor agonist BBN7-14 and antagonist RM26. Bioconjug. Chem. 29, 410–419. 10.1021/acs.bioconjchem.7b00726 29254329 PMC5824342

[B26] Coutinho I. Day T. K. Tilley W. D. Selth L. A. (2016). Androgen receptor signaling in castration-resistant prostate cancer: a lesson in persistence. Endocr. Relat. Cancer 23, T179–T197. 10.1530/ERC-16-0422 27799360

[B27] Coy D. H. Heinz-Erian P. Jiang N. Y. Sasaki Y. Taylor J. Moreau J. P.. (1988). Probing peptide backbone function in bombesin. a reduced peptide bond analogue with potent and specific receptor antagonist activity. J. Biol. Chem. 263, 5056–5060. 2451661

[B28] Dall'oglio M. F. Crippa A. Antunes A. A. Nesrallah L. J. Leite K. R. Srougi M. (2005). Survival of patients with prostate cancer and normal PSA levels treated by radical prostatectomy. Int. Braz. J. Urol. 31, 222–227. 10.1590/S1677-55382005000300005 15992424

[B29] Dalm S. U. Bakker I. L. de Blois E. Doeswijk G. N. Konijnenberg M. W. Orlandi F.. (2017). ^68^Ga/177Lu-NeoBOMB1, a novel radiolabeled GRPR antagonist for theranostic use in oncology. J. Nucl. Med. 58, 293–299. 10.2967/jnumed.116.176636 27609789

[B30] DaSilva J. O. Amorino G. P. Casarez E. V. Pemberton B. Parsons S. J. (2013). Neuroendocrine-derived peptides promote prostate cancer cell survival through activation of IGF-1R signaling. Prostate 73, 801–812. 10.1002/pros.22624 23192379 PMC4085781

[B31] De Vincentis G. Remediani S. Varvarigou A. D. Di Santo G. Iori F. Laurenti C.. (2004). Role of 99mTc-bombesin scan in diagnosis and staging of prostate cancer. Cancer Biother. Radiopharm. 19, 81–84. 10.1089/108497804773391711 15068615

[B32] Deng H. Wang H. Zhang H. Wang M. Giglio B. Ma X.. (2017). Imaging neurotensin receptor in prostate cancer with (64)Cu-labeled neurotensin analogs. Mol. Imaging 16:1536012117711369. 10.1177/1536012117711369 28849698 PMC6081756

[B33] DeSantis C. E. Miller K. D. Dale W. Mohile S. G. Cohen H. J. Leach C. R.. (2019). Cancer statistics for adults aged 85 years and older, 2019. 69, 452–467. 10.3322/caac.21577 31390062 PMC12103238

[B34] di Sant'Agnese P. A. (2001). Neuroendocrine differentiation in prostatic carcinoma: an update on recent developments. Ann. Oncol. 12, S135–S140. 10.1093/annonc/12.suppl_2.S135 11762341

[B35] Diao W. Cai H. Chen L. Jin X. Liao X. Jia Z. (2019). Recent advances in prostate-specific membrane antigen-based radiopharmaceuticals. Curr. Top. Med. Chem. 19, 33–56. 10.2174/1568026619666190201100739 30706785

[B36] Dos Santos G. Garcia Fontes M. Engler H. Alonso O. (2018). Intraindividual comparison of ^68^Ga-DOTATATE PET / CT vs (11)C-Choline PET / CT in patients with prostate cancer in biochemical relapse: *in vivo* evaluation of the expression of somatostatin receptors. Rev. Esp. Med. Nucl. Imagen. Mol. 38:29–37. 10.1016/j.remnie.2018.11.008 30442558

[B37] Dos Santos G. García Fontes M. Engler H. Alonso O. (2019). Intraindividual comparison of (68)Ga-DOTATATE PET / CT vs (11)C-Choline PET / CT in patients with prostate cancer in biochemical relapse: *in vivo* evaluation of the expression of somatostatin receptors. Rev. Esp. Med. Nucl. Imagen. Mol. 38, 29–37. 10.1016/j.remn.2018.08.005 30442558

[B38] Durkan K. Jiang Z. Rold T. L. Sieckman G. L. Hoffman T. J. Bandari R. P.. (2014). A heterodimeric [RGD-Glu-[^64^Cu-NO2A]-6-Ahx-RM2] alphavbeta3/GRPr-targeting antagonist radiotracer for PET imaging of prostate tumors. Nucl. Med. Biol. 41, 133–139. 10.1016/j.nucmedbio.2013.11.006 24480266 PMC4022290

[B39] Eder M. Schafer M. Bauder-Wust U. Haberkorn U. Eisenhut M. Kopka K. (2014). Preclinical evaluation of a bispecific low-molecular heterodimer targeting both PSMA and GRPR for improved PET imaging and therapy of prostate cancer. Prostate 74, 659–668. 10.1002/pros.22784 24464532

[B40] Elshafae S. M. Hassan B. B. Supsavhad W. Dirksen W. P. Camiener R. Y. Ding H.. (2016). Gastrin-releasing peptide receptor (GRPr) promotes EMT, growth, and invasion in canine prostate cancer. Prostate 76, 796–809. 10.1002/pros.23154 26939805 PMC5867904

[B41] Elshan N. Rettig M. B. Jung M. E. (2018). Molecules targeting the androgen receptor (AR) signaling axis beyond the AR-Ligand binding domain. Med. Res. Rev. 39, 910–960. 10.1002/med.21548 30565725 PMC6608750

[B42] Evans B. J. King A. T. Katsifis A. Matesic L. Jamie J. F. (2020). Methods to enhance the metabolic stability of peptide-based PET radiopharmaceuticals. Molecules 25:2314. 10.3390/molecules25102314 32423178 PMC7287708

[B43] Fassbender T. F. Schiller F. Mix M. Maecke H. R. Kiefer S. Drendel V.. (2019). Accuracy of [^68^Ga]Ga-RM2-PET/CT for diagnosis of primary prostate cancer compared to histopathology. Nucl. Med. Biol. 70, 32–38. 10.1016/j.nucmedbio.2019.01.009 30836254

[B44] Fassbender T. F. Schiller F. Zamboglou C. Drendel V. Kiefer S. Jilg C. A.. (2020). Voxel-based comparison of [(68)Ga]Ga-RM2-PET/CT and [(68)Ga]Ga-PSMA-11-PET/CT with histopathology for diagnosis of primary prostate cancer. EJNMMI Res. 10:62. 10.1186/s13550-020-00652-y 32533273 PMC7292851

[B45] Ferreira C. A. Fuscaldi L. L. Townsend D. M. Rubello D. A. Barros L. B. (2017). Radiolabeled bombesin derivatives for preclinical oncological imaging. Biomed. Pharmacother. 87, 58–72. 10.1016/j.biopha.2016.12.083 28040598 PMC5331929

[B46] Fischer G. Lindner S. Litau S. Schirrmacher R. Wangler B. Wangler C. (2015). Next step toward optimization of GRP receptor avidities: determination of the minimal distance between BBN(7-14) units in peptide homodimers. Bioconjug. Chem. 26, 1479–1483. 10.1021/acs.bioconjchem.5b00362 26200324

[B47] Ghosh A. Raju N. Tweedle M. Kumar K. (2017). *In vitro* mouse and human serum stability of a heterobivalent dual-target probe that has strong affinity to gastrin-releasing peptide and neuropeptide Y1 receptors on tumor cells. Cancer Biother. Radiopharm. 32, 24–32. 10.1089/cbr.2016.2136 28186846 PMC5911699

[B48] Giovacchini G. Giovannini E. Riondato M. Ciarmiello A. (2017). Radiopharmaceuticals for the diagnosis and therapy of neuroendocrine differentiated prostate cancer. Curr. Radiopharm. 10, 6–15. 10.2174/1874471009666161229123126 28034291

[B49] Gofrit O. N. Frank S. Meirovitz A. Nechushtan H. Orevi M. (2017). PET/CT with 68Ga-DOTA-TATE for diagnosis of neuroendocrine: differentiation in patients with castrate-resistant prostate cancer. Clin. Nucl. Med. 42, 1–6. 10.1097/RLU.0000000000001424 27775942

[B50] Gourni E. Del Pozzo L. Kheirallah E. Smerling C. Waser B. Reubi J. C.. (2015). Copper-64 labeled macrobicyclic sarcophagine coupled to a GRP receptor antagonist shows great promise for PET imaging of prostate cancer. Mol. Pharm. 12, 2781–2790. 10.1021/mp500671j 26132879

[B51] Gourni E. Mansi R. Jamous M. Waser B. Smerling C. Burian A.. (2014). N-terminal modifications improve the receptor affinity and pharmacokinetics of radiolabeled peptidic gastrin-releasing peptide receptor antagonists: examples of 68Ga- and 64Cu-labeled peptides for PET imaging. J. Nucl. Med. 55, 1719–1725. 10.2967/jnumed.114.141242 25146125

[B52] Graham M. M. Menda Y. (2011). Radiopeptide imaging and therapy in the United States. J. Nucl. Med. 52(Suppl 2), 56s−63s. 10.2967/jnumed.110.085746 22144556

[B53] Ho C. L. Liu I. H. Wu Y. H. Chen L. C. Chen C. L. Lee W. C.. (2011). Molecular imaging, pharmacokinetics, and dosimetry of in-AMBA in human prostate tumor-bearing mice. J. Biomed. Biotechnol. 2011:101497. 10.1155/2011/101497 21660132 PMC3110286

[B54] Iagaru A. (2017). Will GRPR compete with PSMA as a target in prostate cancer,? J. Nucl. Med. 58, 1883–1884. 10.2967/jnumed.117.198192 28970333

[B55] Kahkonen E. Jambor I. Kemppainen J. Lehtio K. Gronroos T. J. Kuisma A.. (2013). *In vivo* imaging of prostate cancer using [^68^Ga]-labeled bombesin analog BAY86-7548. Clin. Cancer Res. 19, 5434–5443. 10.1158/1078-0432.CCR-12-3490 23935037

[B56] Kaloudi A. Lymperis E. Giarika A. Dalm S. Orlandi F. Barbato D.. (2017). NeoBOMB1, a GRPR-antagonist for breast cancer theragnostics: first results of a preclinical Study with [^67^Ga]NeoBOMB1 in T-47D cells and tumor-bearing mice. Molecules 22:1950. 10.3390/molecules22111950 29137110 PMC6150197

[B57] Kamaleshwaran K. K. Joseph J. Upadhya I. Shinto A. S. (2017). Image findings of a rare case of neuroendocrine tumor metastatic to orbital extraocular muscle in gallium-68 dotanoc positron emission tomography/computed tomography and therapy with lutetium-177 dotatate. Ind. J. Nucl. Med. 32, 125–127. 10.4103/0972-3919.202236 28533641 PMC5439203

[B58] Kim M. H. Park J. A. Woo S. K. Lee K. C. An G. I. Kim B. S.. (2015). Evaluation of a (6)(4)Culabeled 1,4,7triazacyclononane, 1glutaric acid4,7 acetic acid (NODAGA)galactosebombesin analogue as a PET imaging probe in a gastrinreleasing peptide receptorexpressing prostate cancer xenograft model. Int. J. Oncol. 46, 1159–1168. 10.3892/ijo.2015.2832 25586565

[B59] Korner M. Waser B. Rehmann R. Reubi J. C. (2014). Early over-expression of GRP receptors in prostatic carcinogenesis. Prostate 74, 217–224. 10.1002/pros.22743 24150752

[B60] Koutsilieris M. Dimopoulos T. Milathianakis C. Bogdanos J. Karamanolakis D. Pissimissis N.. (2007). Combination of somatostatin analogues and dexamethasone (anti-survival-factor concept) with luteinizing hormone-releasing hormone in androgen ablation-refractory prostate cancer with bone metastasis. BJU Int. 100(Suppl. 2):60–62. 10.1111/j.1464-410X.2007.06958.x 17594363

[B61] Kurth J. Krause B. J. Schwarzenböck S. M. Bergner C. Hakenberg O. W. Heuschkel M. (2020). First-in-human dosimetry of gastrin-releasing peptide receptor antagonist [(177)Lu]Lu-RM2, a radiopharmaceutical for the treatment of metastatic castration-resistant prostate cancer. Eur. J. Nucl. Med. Mol. Imaging 47, 123–135. 10.1007/s00259-019-04504-3 31482426

[B62] Kwekkeboom D. J. Kooij P. P. Bakker W. H. Macke H. R. Krenning E. P. (1999). Comparison of 111In-DOTA-Tyr3-octreotide and 111In-DTPA-octreotide in the same patients: biodistribution, kinetics, organ and tumor uptake. J. Nucl. Med. 40, 762–767. 10319747

[B63] Lane S. R. Nanda P. Rold T. L. Sieckman G. L. Figueroa S. D. Hoffman T. J.. (2010). Optimization, biological evaluation and microPET imaging of copper-64-labeled bombesin agonists, [64Cu-NO2A-(X)-BBN(7-14)NH2], in a prostate tumor xenografted mouse model. Nucl. Med. Biol. 37, 751–761. 10.1016/j.nucmedbio.2010.04.016 20870150

[B64] Lenzo N. Meyrick D. J. Turner J. D. (2018). Review of gallium-68 PSMA PET/CT imaging in the management of prostate cancer. Diagnostics 8:16. 10.3390/diagnostics8010016 29439481 PMC5871999

[B65] Li P. Chen J. Kashiwagi E. Mizushima T. Han B. Inoue S.. (2018). The interaction between androgen receptor and semenogelin I: a synthetic LxxLL peptide antagonist inhibits the growth of prostate cancer cells. Br. J. Cancer 118, 416–420. 10.1038/bjc.2017.404 29136406 PMC5808024

[B66] Lindner S. Fiedler L. Wangler B. Bartenstein P. Schirrmacher R. Wangler C. (2018). Design, synthesis and *in vitro* evaluation of heterobivalent peptidic radioligands targeting both GRP- and VPAC1-Receptors concomitantly overexpressed on various malignancies - is the concept feasible? Eur. J. Med. Chem. 155, 84–95. 10.1016/j.ejmech.2018.05.047 29864700

[B67] Liolios C. Buchmuller B. Bauder-Wust U. Schafer M. Leotta K. Haberkorn U.. (2018). Monomeric and dimeric (68)Ga-labeled bombesin analogues for positron emission tomography (PET) imaging of tumors expressing gastrin-releasing peptide receptors (GRPrs). J. Med. Chem. 61, 2062–2074. 10.1021/acs.jmedchem.7b01856 29432691

[B68] Liolios C. Schafer M. Haberkorn U. Eder M. Kopka K. (2016). Novel bispecific PSMA/GRPr targeting radioligands with optimized pharmacokinetics for improved pet imaging of prostate cancer. Bioconjug. Chem. 27, 737–751. 10.1021/acs.bioconjchem.5b00687 26726823

[B69] Liu Y. (2006). Radiolabelled somatostatin analog therapy in prostate cancer: current status and future directions. Cancer Lett. 239, 21–26. 10.1016/j.canlet.2005.07.020 16126334

[B70] Luboldt W. Zophel K. Wunderlich G. Abramyuk A. Luboldt H. J. Kotzerke J. (2010). Visualization of somatostatin receptors in prostate cancer and its bone metastases with Ga-68-DOTATOC PET/CT. Mol. Imaging Biol. 12, 78–84. 10.1007/s11307-009-0230-3 19421819

[B71] Lucente E. Liu H. Liu Y. Hu X. Lacivita E. Leopoldo M.. (2018). Novel ^64^Cu labeled RGD(2)-BBN heterotrimers for PET imaging of prostate cancer. Bioconjug. Chem. 29, 1595–1604. 10.1021/acs.bioconjchem.8b00113 29587479

[B72] Lucia Tornesello A. Lina Tornesello M. Buonaguro F. M. (2017). An overview of bioactive peptides for *in vivo* imaging and therapy in human diseases. Mini Rev. Med. Chem. 17, 758–770. 10.2174/1389557517666170120151739 28117023

[B73] Lütje S. Heskamp S. Cornelissen A. S. Poeppel T. D. van den Broek S. A. Rosenbaum-Krumme S. Bockisch A.. (2015). PSMA ligands for radionuclide imaging and therapy of prostate cancer: clinical status. Theranostics 5:1388. 10.7150/thno.13348 26681984 PMC4672020

[B74] Lymperis E. Kaloudi A. Sallegger W. Bakker I. L. Krenning E. P. de Jong M.. (2018). Radiometal-dependent biological profile of the radiolabeled gastrin-releasing peptide receptor antagonist SB3 in cancer theranostics: metabolic and biodistribution patterns defined by neprilysin. Bioconjug. Chem. 29, 1774–1784. 10.1021/acs.bioconjchem.8b00225 29664606

[B75] Maddalena M. E. Fox J. Chen J. Feng W. Cagnolini A. Linder K. E.. (2009). 177Lu-AMBA biodistribution, radiotherapeutic efficacy, imaging, and autoradiography in prostate cancer models with low GRP-R expression. J. Nucl. Med. 50, 2017–2024. 10.2967/jnumed.109.064444 19910427

[B76] Maffioli L. Florimonte L. Costa D. C. Correia Castanheira J. Grana C. Luster M.. (2015). New radiopharmaceutical agents for the treatment of castration-resistant prostate cancer. Q. J. Nucl. Med. Mol. Imaging 59, 420–438. 26222274

[B77] Maina T. Bergsma H. Kulkarni H. R. Mueller D. Charalambidis D. Krenning E. P.. (2016). Preclinical and first clinical experience with the gastrin-releasing peptide receptor-antagonist Ga-68 SB3 and PET/CT. Eur. J. Nucl. Med. Mol. Imaging 43, 964–973. 10.1007/s00259-015-3232-1 26631238

[B78] Maina T. Kaloudi A. Valverde I. E. Mindt T. L. Nock B. A. (2017a). Amide-to-triazole switch vs. *in vivo* NEP-inhibition approaches to promote radiopeptide targeting of GRPR-positive tumors. Nucl. Med. Biol. 52, 57–62. 10.1016/j.nucmedbio.2017.06.001 28636973

[B79] Maina T. Nock B. A. (2017). From bench to bed: new gastrin-releasing peptide receptor-directed radioligands and their use in prostate cancer. PET Clin. 12, 205–217. 10.1016/j.cpet.2016.12.002 28267454

[B80] Maina T. Nock B. A. Kulkarni H. Singh A. Baum R. P. (2017b). Theranostic prospects of gastrin-releasing peptide receptor-radioantagonists in oncology. PET Clin. 12, 297–309. 10.1016/j.cpet.2017.02.007 28576168

[B81] Mansi R. Fleischmann A. Macke H. R. Reubi J. C. (2013). Targeting GRPR in urological cancers–from basic research to clinical application. Nat. Rev. Urol. 10, 235–244. 10.1038/nrurol.2013.42 23507930

[B82] Mansi R. Wang X. Forrer F. Kneifel S. Tamma M. L. Waser B.. (2009). Evaluation of a 1,4,7,10-tetraazacyclododecane-1,4,7,10-tetraacetic acid-conjugated bombesin-based radioantagonist for the labeling with single-photon emission computed tomography, positron emission tomography, and therapeutic radionuclides. Clin. Cancer Res. 15, 5240–5249. 10.1158/1078-0432.CCR-08-3145 19671861

[B83] Mansi R. Wang X. Forrer F. Waser B. Cescato R. Graham K.. (2011). Development of a potent DOTA-conjugated bombesin antagonist for targeting GRPr-positive tumours. Eur. J. Nucl. Med. Mol. Imaging 38, 97–107. 10.1007/s00259-010-1596-9 20717822

[B84] Mansour N. Paquette M. Ait-Mohand S. Dumulon-Perreault V. Guerin B. (2018). Evaluation of a novel GRPR antagonist for prostate cancer PET imaging: [^64^Cu]-DOTHA2-PEG-RM26. Nucl. Med. Biol. 56, 31–38. 10.1016/j.nucmedbio.2017.10.006 29154145

[B85] Matei D. V. Renne G. Pimentel M. Sandri M. T. Zorzino L. Botteri E.. (2012). Neuroendocrine differentiation in castration-resistant prostate cancer: a systematic diagnostic attempt. Clin. Genitourin. Cancer 10, 164–173. 10.1016/j.clgc.2011.12.004 22401754

[B86] Mather S. J. (2007). Design of radiolabelled ligands for the imaging and treatment of cancer. Mol. Biosyst. 3, 30–35. 10.1039/B611736H. 17216053

[B87] Minamimoto R. Hancock S. Schneider B. Chin F. T. Jamali M. Loening A.. (2016). Pilot comparison of ^68^Ga-RM2 PET and ^68^Ga-PSMA-11 PET in patients with biochemically recurrent prostate cancer. J. Nucl. Med. 57, 557–562. 10.2967/jnumed.115.168393 26659347

[B88] Minamimoto R. Sonni I. Hancock S. Vasanawala S. Loening A. Gambhir S. S.. (2018). Prospective evaluation of (68)Ga-RM2 PET/MRI in patients with biochemical recurrence of prostate cancer and negative findings on conventional imaging. J. Nucl. Med. 59, 803–808. 10.2967/jnumed.117.197624 29084827

[B89] Mitran B. Thisgaard H. Rinne S. Dam J. H. Azami F. Tolmachev V.. (2019). Selection of an optimal macrocyclic chelator improves the imaging of prostate cancer using cobalt-labeled, GRPR antagonist RM26. Sci. Rep. 9:17086. 10.1038/s41598-019-52914-y 31745219 PMC6863848

[B90] Mitran B. Thisgaard H. Rosenstrom U. Dam J. H. Larhed M. Tolmachev V.. (2017). High contrast PET imaging of GRPR expression in prostate cancer using cobalt-labeled bombesin antagonist RM26. Contrast Media Mol. Imaging 2017:6873684. 10.1155/2017/6873684 29097932 PMC5612608

[B91] Mitran B. Varasteh Z. Selvaraju R. K. Lindeberg G. Sorensen J. Larhed M.. (2016). Selection of optimal chelator improves the contrast of GRPR imaging using bombesin analogue RM26. Int. J. Oncol. 48, 2124–2134. 10.3892/ijo.2016.3429 26983776

[B92] Mohtavinejad N. Shafiee Ardestani M. Khalaj A. Pormohammad A. Najafi R. Bitarafan-Rajabi A.. (2020). Application of radiolabeled peptides in tumor imaging and therapy. Life Sci. 258:118206. 10.1016/j.lfs.2020.118206 32758623

[B93] Moreno P. Ramos-Alvarez I. Moody T. W. Jensen R. T. (2016). Bombesin related peptides/receptors and their promising therapeutic roles in cancer imaging, targeting and treatment. Expert Opin. Ther. Targets 20, 1055–1073. 10.1517/14728222.2016.1164694 26981612 PMC5067074

[B94] Morgat C. Mishra A. K. Varshney R. Allard M. Fernandez P. Hindie E. (2014). Targeting neuropeptide receptors for cancer imaging and therapy: perspectives with bombesin, neurotensin, and neuropeptide-Y receptors. J. Nucl. Med. 55, 1650–1657. 10.2967/jnumed.114.142000 25189338

[B95] Nilsson S. Reubi J. C. Kalkner K. M. Laissue J. A. Horisberger U. Olerud C. (1995). metastatic hormone-refractory prostatic adenocarcinoma expresses somatostatin receptors and is visualized *in-vivo* by in-111 -labeled dtpa-d- phe(1) -octreotide scintigraph. Cancer Res. 55, S5805–S5810. 7493350

[B96] Nock B. A. Kaloudi A. Lymperis E. Giarika A. Kulkarni H. R. Klette I.. (2017). Theranostic perspectives in prostate cancer with the gastrin-releasing peptide receptor antagonist neobomb1, preclinical and first clinical results. J. Nucl. Med. 58, 75–80. 10.2967/jnumed.116.178889 27493272

[B97] Parida G. K. Tripathy S. Datta Gupta S. Singhal A. Kumar R. Bal C.. (2018). Adenocarcinoma prostate with neuroendocrine differentiation: potential utility of 18F-FDG PET/CT and 68Ga-DOTANOC PET/CT Over 68Ga-PSMA PET/CT. Clin. Nucl. Med. 43, 248–249. 10.1097/RLU.0000000000002013 29474196

[B98] Pourghiasian M. Liu Z. Pan J. Zhang Z. Colpo N. Lin K. S.. (2015). (18)F-AmBF3-MJ9, a novel radiofluorinated bombesin derivative for prostate cancer imaging. Bioorg. Med. Chem. 23, 1500–1506. 10.1016/j.bmc.2015.02.009 25757604

[B99] Pressly E. D. Pierce R. A. Connal L. A. Hawker C. J. Liu Y. (2013). Nanoparticle PET/CT imaging of natriuretic peptide clearance receptor in prostate cancer. Bioconjug. Chem. 24, 196–204. 10.1021/bc300473x 23272904 PMC3578065

[B100] Qiao J. Grabowska M. M. Forestier-Roman I. S. Mirosevich J. Case T. C. Chung D. H.. (2016). Activation of GRP/GRP-R signaling contributes to castration-resistant prostate cancer progression. Oncotarget 7, 61955–61969. 10.18632/oncotarget.11326 27542219 PMC5308703

[B101] Sah B. R. Burger I. A. Schibli R. Friebe M. Dinkelborg L. Graham K.. (2015). Dosimetry and first clinical evaluation of the new 18F-radiolabeled bombesin analogue BAY 864367 in patients with prostate cancer. J. Nucl. Med. 56, 372–378. 10.2967/jnumed.114.147116 25678494

[B102] Savelli G. Muni A. Falchi R. Zaniboni A. Barbieri R. Valmadre G.. (2015). Somatostatin receptors over-expression in castration resistant prostate cancer detected by PET/CT: preliminary report of in six patients. Ann. Transl. Med. 3:145. 10.3978/j.issn.2305-5839.2015.06.10 26207238 PMC4486921

[B103] Schottelius M. Wester H. J. (2009). Molecular imaging targeting peptide receptors. Methods 48, 161–177. 10.1016/j.ymeth.2009.03.012 19324088

[B104] Schroeder R. P. van Weerden W. M. Bangma C. Krenning E. P. de Jong M. (2009). Peptide receptor imaging of prostate cancer with radiolabelled bombesin analogues. Methods 48, 200–204. 10.1016/j.ymeth.2009.04.002 19398012

[B105] Schuster D. M. Nanni C. Fanti S. (2016). PET tracers beyond FDG in prostate cancer. Semin. Nucl. Med. 46, 507–521. 10.1053/j.semnuclmed.2016.07.005 27825431 PMC5117950

[B106] Scopinaro F. De Vincentis G. Varvarigou A. D. Laurenti C. Iori F. Remediani S.. (2003). 99mTc-bombesin detects prostate cancer and invasion of pelvic lymph nodes. Eur. J. Nucl. Med. Mol. Imaging 30, 1378–1382. 10.1007/s00259-003-1261-7 12920485

[B107] Siegel R. L. Miller K. D. Jemal A. (2020). Cancer statistics, 2020. CA Cancer J. Clin. 70, 7–30. 10.3322/caac.21590 31912902

[B108] Siegel R. L. Miller K. D. Jemal A. J. C. (2019). Cancer statistics, 2019. 69, 7–34. 10.3322/caac.21551 30620402

[B109] Silver D. A. Pellicer I. Fair W. R. Heston W. D. Cordon-Cardo C. (1997). Prostate-specific membrane antigen expression in normal and malignant human tissues. Clin. Cancer Res. 3, 81–85. 9815541

[B110] Singh H. Canto E. I. Shariat S. F. Kadmon D. Miles B. J. Wheeler T. M.. (2004). Predictors of prostate cancer after initial negative systematic 12 core biopsy. J. Urol. 171, 1850–1854. 10.1097/01.ju.0000119667.86071.e7 15076292

[B111] Smith C. J. Volkert W. A. Hoffman T. J. (2005). Radiolabeled peptide conjugates for targeting of the bombesin receptor superfamily subtypes. Nucl. Med. Biol. 32, 733–740. 10.1016/j.nucmedbio.2005.05.005 16243649

[B112] Spieth M. E. Lin Y. G. Nguyen T. T. (2002). Diagnosing and treating small-cell carcinomas of prostatic origin. Clin. Nucl. Med. 27, 11–17. 10.1097/00003072-200201000-00003 11805477

[B113] Stott Reynolds T. J. Schehr R. Liu D. Xu J. Miao Y. Hoffman T. J.. (2015). Characterization and evaluation of DOTA-conjugated Bombesin/RGD-antagonists for prostate cancer tumor imaging and therapy. Nucl. Med. Biol. 42, 99–108. 10.1016/j.nucmedbio.2014.10.002 25459113

[B114] Stott Reynolds T. J. Smith C. J. Lewis M. R. (2018). Peptide-based radiopharmaceuticals for molecular imaging of prostate cancer. Adv. Exp. Med. Biol. 1096, 135–158. 10.1007/978-3-319-99286-0_8 30324352

[B115] Sun X. Li Y. Liu T. Li Z. Zhang X. Chen X. (2017). Peptide-based imaging agents for cancer detection. Adv. Drug Deliv. Rev. 110–111, 38–51. 10.1016/j.addr.2016.06.007 27327937 PMC5235994

[B116] Sun Y. Ma X. Zhang Z. Sun Z. Loft M. Ding B.. (2016). Preclinical study on GRPR-Targeted (68)Ga-probes for PET imaging of prostate cancer. Bioconjug. Chem. 27, 1857–1864. 10.1021/acs.bioconjchem.6b00279 27399868

[B117] Taylor R. M. Severns V. Brown D. C. Bisoffi M. Sillerud L. O. (2012). Prostate cancer targeting motifs: expression of alphanu beta3, neurotensin receptor 1, prostate specific membrane antigen, and prostate stem cell antigen in human prostate cancer cell lines and xenografts. Prostate 72, 523–532. 10.1002/pros.21454 21748756 PMC4366051

[B118] Thakur M. L. Kolan H. Li J. Wiaderkiewicz R. Pallela V. R. Duggaraju R.. (1997). Radiolabeled somatostatin analogs in prostate cancer. Nucl. Med. Biol. 24, 105–113. 10.1016/S0969-8051(96)00180-1 9080482

[B119] Tornesello A. L. Buonaguro L. Tornesello M. L. Buonaguro F. M. (2017). New insights in the design of bioactive peptides and chelating agents for imaging and therapy in oncology. Molecules 22:1282. 10.3390/molecules22081282 28767081 PMC6152110

[B120] Touijer K. A. Michaud L. H. Alvarez A. V. Gopalan A. Kossatz S. Gonen M.. (2019). Prospective study of the radiolabeled GRPR antagonist BAY86-7548 for positron emission tomography/computed tomography imaging of newly diagnosed prostate cancer. Eur. Urol. Oncol. 2, 166–173. 10.1016/j.euo.2018.08.011 31017093 PMC7480883

[B121] Usmani S. Ahmed N. Marafi F. Rasheed R. Amanguno H. G. Al Kandari F.. (2017). Clin. Nucl. Med. 42, 410–413. 10.1097/RLU.0000000000001618 28240661

[B122] Usmani S. Orevi M. Stefanelli A. Zaniboni A. Gofrit O. N. Bn,à C.. (2019). Neuroendocrine differentiation in castration resistant prostate cancer. Nuclear medicine radiopharmaceuticals and imaging techniques: a narrative review. Crit. Rev. Oncol. Hematol. 138, 29–37. 10.1016/j.critrevonc.2019.03.005 31092382

[B123] Valverde I. E. Huxol E. Mindt T. L. (2014). Radiolabeled antagonistic bombesin peptidomimetics for tumor targeting. J. Labelled Comp. Radiopharm. 57, 275–278. 10.1002/jlcr.3162 24327435

[B124] Valverde I. E. Vomstein S. Fischer C. A. Mascarin A. Mindt T. L. (2015). Probing the backbone function of tumor targeting peptides by an amide-to-triazole substitution strategy. J Med. Chem. 58, 7475–7484. 10.1021/acs.jmedchem.5b00994 26309061

[B125] Valverde I. E. Vomstein S. Mindt T. L. (2016). Toward the optimization of bombesin-based radiotracers for tumor targeting. J. Med. Chem. 59, 3867–3877. 10.1021/acs.jmedchem.6b00025 27054526

[B126] Van de Wiele C. Dumont F. Dierckx R. A. Peers S. H. Thornback J. R. Slegers G. Thierens H. (2001). Biodistribution and dosimetry of (99m)Tc-RP527, a gastrin-releasing peptide (GRP) agonist for the visualization of GRP receptor-expressing malignancies. J. Nucl. Med. 42, 1722–1727. 10.1097/00006231-200006000-00078 11696645

[B127] Van de Wiele C. Dumont F. Vanden Broecke R. Oosterlinck W. Cocquyt V. Serreyn R.. (2000). Technetium-99m RP527, a GRP analogue for visualisation of GRP receptor-expressing malignancies: a feasibility study. Eur. J. Nucl. Med. 27, 1694–1699. 10.1007/s002590000355 11105826

[B128] Van Poppel H. Vekemans K. Da Pozzo L. Bono A. Kliment J. Montironi R.. (2006). Radical prostatectomy for locally advanced prostate cancer: results of a feasibility study (EORTC 30001). Eur. J. Cancer 42, 1062–1067. 10.1016/j.ejca.2005.11.030 16624554

[B129] Varasteh Z. Aberg O. Velikyan I. Lindeberg G. Sorensen J. Larhed M.. (2013). *In vitro* and *in vivo* evaluation of a (18)F-labeled high affinity NOTA conjugated bombesin antagonist as a PET ligand for GRPR-targeted tumor imaging. PLoS ONE 8:e81932. 10.1371/journal.pone.0081932 24312607 PMC3849266

[B130] Varasteh Z. Mitran B. Rosenstrom U. Velikyan I. Rosestedt M. Lindeberg G.. (2015). The effect of macrocyclic chelators on the targeting properties of the 68Ga-labeled gastrin releasing peptide receptor antagonist PEG2-RM26. Nucl. Med. Biol. 42, 446–454. 10.1016/j.nucmedbio.2014.12.009 25684649

[B131] Varasteh Z. Rosenstrom U. Velikyan I. Mitran B. Altai M. Honarvar H.. (2014). The effect of mini-PEG-based spacer length on binding and pharmacokinetic properties of a 68Ga-labeled NOTA-conjugated antagonistic analog of bombesin. Molecules 19, 10455–10472. 10.3390/molecules190710455 25036155 PMC6270800

[B132] Vargas H. A. Grimm J. Donati F. O. Sala E. Hricak H. (2015). Molecular imaging of prostate cancer: translating molecular biology approaches into the clinical realm. Eur. Radiol. 25, 1294–1302. 10.1007/s00330-014-3539-5 25693661 PMC4994516

[B133] Virgolini I. Szilvasi I. Kurtaran A. Angelberger P. Raderer M. Havlik E.. (1998). Indium-111-DOTA-lanreotide: biodistribution, safety and radiation absorbed dose in tumor patients. J. Nucl. Med. 39, 1928–1936. 9829585

[B134] Wang X. Raulji P. Mohapatra S. S. Patel R. Hellermann G. Kong X.. (2011). Natriuretic peptide receptor a as a novel target for prostate cancer. Mol. Cancer 10:56. 10.1186/1476-4598-10-56 21586128 PMC3121714

[B135] Warrington J. I. Richards G. O. Wang N. (2017). The Role of the Calcitonin Peptide Family in Prostate Cancer and Bone Metastasis. Curr. Mol. Biol. Rep. 3, 197–203. 10.1007/s40610-017-0071-9 28845385 PMC5550541

[B136] Weber W. A. (2014). PET/MR imaging: a critical appraisal. J. Nucl. Med. 55, 56s−58s. 10.2967/jnumed.113.129270 24819418

[B137] Wibmer A. G. Burger I. A. Sala E. Hricak H. Weber W. A. Vargas H. A. (2016). Molecular imaging of prostate cancer. Radiographics 36, 142–159. 10.1148/rg.2016150059 26587888 PMC5410962

[B138] Wieser G. Mansi R. Grosu A. L. Schultze-Seemann W. Dumont-Walter R. A. Meyer P. T.. (2014). Positron emission tomography (PET) imaging of prostate cancer with a gastrin releasing peptide receptor antagonist–from mice to men. Theranostics 4, 412–419. 10.7150/thno.7324 24578724 PMC3936293

[B139] Wieser G. Popp I. Christian Rischke H. Drendel V. Grosu A. L. Bartholoma M.. (2017). Diagnosis of recurrent prostate cancer with PET/CT imaging using the gastrin-releasing peptide receptor antagonist ^68^Ga-RM2, preliminary results in patients with negative or inconclusive [^18^F]Fluoroethylcholine-PET/CT. Eur. J. Nucl. Med. Mol. Imaging 44, 1463–1472. 10.1007/s00259-017-3702-8 28417160

[B140] Yoo S. Kim J. K. Jeong I. G. (2015). Multiparametric magnetic resonance imaging for prostate cancer: a review and update for urologists. Korean J. Urol. 56, 487–497. 10.4111/kju.2015.56.7.487 26175867 PMC4500805

[B141] Zamora P. O. Gulhke S. Bender H. Diekmann D. Rhodes B. A. Biersack H. J.. (1996). Experimental radiotherapy of receptor-positive human prostate adenocarcinoma with 188Re-RC-160, a directly-radiolabeled somatostatin analogue. Int. J. Cancer 65, 214–220. 10.1002/(SICI)1097-0215(19960117)65:2<214::AID-IJC15>3.0.CO;2-D 8567120

[B142] Zamora P. O. Marek M. J. Knapp F. F. Jr. (1997). Preparation of 188Re-RC-160 somatostatin analog: a peptide for local/regional radiotherapy. Appl. Radiat. Isot. 48, 305–309. 10.1016/S0969-8043(96)00226-6 9116651

[B143] Zhang H. Chen J. Waldherr C. Hinni K. Waser B. Reubi J. C.. (2004). Synthesis and evaluation of bombesin derivatives on the basis of pan-bombesin peptides labeled with indium-111, lutetium-177, and yttrium-90 for targeting bombesin receptor-expressing tumors. Cancer Res. 64, 6707–6715. 10.1158/0008-5472.CAN-03-3845 15374988

[B144] Zhang J. Niu G. Fan X. Lang L. Hou G. Chen L.. (2018). PET using a GRPR antagonist Ga-68-RM26 in healthy volunteers and prostate cancer patients. J. Nucl. Med. 59, 922–928. 10.2967/jnumed.117.198929 29123014 PMC6004560

[B145] Zhang J. Niu G. Lang L. Li F. Fan X. Yan X.. (2017). Clinical translation of a dual integrin alphavbeta3- and gastrin-releasing peptide receptor-targeting PET radiotracer, 68Ga-BBN-RGD. J. Nucl. Med. 58, 228–234. 10.2967/jnumed.116.177048 27493267 PMC5288740

[B146] Zhang-Yin J. Provost C. Cancel-Tassin G. Rusu T. Penent M. Radulescu C. (2020). A comparative study of peptide-based imaging agents [^68^Ga]Ga-PSMA-11, [^68^Ga]Ga-AMBA, [^68^Ga]Ga-NODAGA-RGD and [^68^Ga]Ga-DOTA-NT-20.3 in preclinical prostate tumour models. Nucl. Med. Biol. 84–85, 88–95. 10.1016/j.nucmedbio.2020.03.005 32251995

[B147] Zhou J. Gao S. Hsieh C. L. Malla M. Shemshedini L. (2017). Peptide B targets soluble guanylyl cyclase alpha1 and kills prostate cancer cells. PLoS ONE 12:e0184088. 10.1371/journal.pone.0184088 28859127 PMC5578680

